# Identification of a Lipid-Exposed Extrahelical Binding Site for Positive Allosteric Modulators of the Dopamine D_2_ Receptor

**DOI:** 10.1021/acschemneuro.5c00105

**Published:** 2025-05-15

**Authors:** Herman D. Lim, Damian Bartuzi, Alastair C Keen, Caroline Rauffenbart, Jacqueline Glenn, Steven J. Charlton, Silvia Lovera, Zara A. Sands, Ali Ates, Martyn Wood, Meritxell Canals, Jonathan A. Javitch, Jens Carlsson, J. Robert Lane

**Affiliations:** 1 Drug Discovery Biology, Monash Institute of Pharmaceutical Sciences, 2541 Monash University (Parkville Campus), 399 Royal Parade, Parkville, VIC 3052, Australia; 2 Science for Life Laboratory, Department of Cell and Molecular Biology, 8097 Uppsala University, Uppsala SE- 751 24, Sweden; 3 Department of Synthesis and Chemical Technology of Pharmaceutical Substances with Computer Modeling Laboratory, Faculty of Pharmacy, Medical University of Lublin, 4A Chodźki St., Lublin 20093, Poland; 4 Division of Physiology, Pharmacology and Neuroscience, School of Life Sciences, Queen’s Medical Centre, 6123 University of Nottingham, Nottingham NG7 2UH, United Kingdom; 5 Centre of Membrane Proteins and Receptors, University of Birmingham and University of Nottingham, Nottingham NG7 2UH, United Kingdom; 6 Departments of Psychiatry and Pharmacology, College of Physicians and Surgeons, 5798 Columbia University, New York, New York 10032, United States; 7 Division of Molecular Therapeutics, New York State Psychiatric Institute, New York, New York 10032, United States; 8 UCB BioPharma SPRL, Chemin de Foriest, Braine-l’Alleud, Brussels B-1420, Belgium

**Keywords:** dopamine D_2_ receptor, G protein-coupled receptor, allosteric site, positive allosteric modulator

## Abstract

Recently, the first small-molecule positive allosteric modulators (PAMs) of the dopamine D_2_ receptor (D_2_R) were identified. The more potent PAM potentiated the effects of D_2_R signaling in vitro and in an in vivo model predictive of anti-Parkinson’s efficacy. We reveal, based on the results of our site-directed mutagenesis and molecular dynamics experiments, that this scaffold binds to a hitherto unexploited lipid-exposed extrahelical allosteric site in the D_2_R that lies in a cleft toward the intracellular aspect of the D_2_R defined by residues in transmembrane domains 1 and 7 and helix 8. By binding to this site, the PAM acts to potentiate the binding affinity of efficacious agonists, such as dopamine. Our simulations suggest that the PAM achieves this effect by stabilizing an active-like conformation of the receptor, similar to the G protein-bound state with TM5 and the tyrosine toggle switch playing the major role.

## Graphical abstract

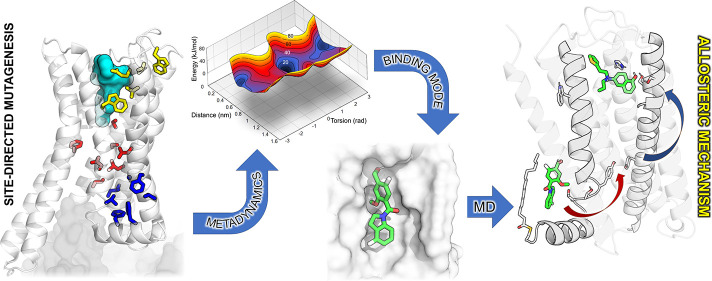

## Introduction

Monoamine dopamine (DA) acts via G protein-coupled dopamine receptors to modulate physiological processes including motor function, reward mechanisms, and the learning process.^1^ This family of five receptors (D_1–5_R) is divided into the G_olf/s_ -coupled D_1_-like receptors (D_1_R and D_5_R) and the D_2_-like (D_2_R, D_3_R, and D_4_R) that couple to the Gα_i/o/z_ family of G proteins to inhibit cAMP production. Pharmacological agents targeting DRs are used clinically in the management of neurological and psychiatric disorders, including Parkinson’s disease (PD).^2,3^ Currently, these drugs target the orthosteric site in the transmembrane crevice where DA binds and, because this site is conserved across DR subtypes, show limited subtype selectivity.

PD is a progressive neurodegenerative disorder epitomized by a loss of dopaminergic neurons from the substantia nigra and is characterized by a profound disruption of motor function.^4,5^ The DA precursor l-3,4-dihydroxyphenylalanine (L-DOPA) remains the standard treatment for PD. Prolonged L-DOPA treatment, however, is associated with abrupt transitions between effective control and a lack of efficacy along with L-DOPA-induced dyskinesias (LIDs).^6^ While the use of DR agonists as monotherapy in early PD patients is associated with lower overall incidence of LIDs, the majority of patients require the addition of L-DOPA 2–5 years from disease onset.^2^ The superior efficacy of L-DOPA compared to that of DR agonists may be because it maintains the phasic pattern of dopamine release in the brain. DR agonists are often associated with side effects that include induction of psychosis, nausea, hypotension, and symptoms of impulse control disorder such as compulsive gambling.^7^ These limiting side effects could be due to a lack of target specificity or the inability to replicate the spatiotemporal pattern of endogenous DA signaling in the brain.

Targeting topographically distinct allosteric sites with small molecules (allosteric modulators) has become the focus of much drug research.^8–10^ There is a higher likelihood of attaining target selectivity because allosteric sites are not as conserved as the orthosteric sites. Allosteric modulators offer the potential for “fine-tuning” of normal physiological signaling because they still allow for the endogenous agonists to bind to the receptor. A D_2_R PAM that acts to potentiate the effect of DA would maintain the temporal and regional aspects of physiological DA signaling, unlike the continuous stimulation associated with a DR agonist. Therefore, a PAM may have an improved side effect profile and, similar to L-DOPA, superior efficacy compared with DR agonists. Furthermore, a PAM may potentiate the effects of a low, non-neurotoxic dose of L-DOPA, thus prolonging the window of effective treatment. Significant challenges remain for the discovery and development of drugs targeting allosteric sites of G protein-coupled receptors (GPCRs).^10^ The binding mode of an allosteric ligand is often unknown, as is the mechanism by which it modulates the binding and function of an orthosteric ligand. This presents a barrier to the progression of allosteric modulators to clinically effective therapeutics and may explain the paucity of drugs in the clinic that act via an allosteric mechanism on a GPCR target.

Recently, a novel benzothiazole compound, 1,3-benzothiazol-2-yl­(2-methyl-2,3-dihydro-indol-1-yl)­methanone (D2 PAM 1, *vide* Supporting Information S1) was reported as the first small-molecule PAM of the D_2_R.^11^ D2 PAM1 potentiated the effects of DA-stimulated D_2_R signaling in vitro and in electrophysiological studies on dissociated striatal neurons. A more potent compound from the series based on this scaffold was identified, [5-fluoro-4-(hydroxymethyl)-2-methoxyphenyl]-(4-fluoro-1*H*-indol-1-yl)­methanone (D2 PAM2, shown in Supporting Information S1).^11^ D2 PAM2 was shown to potentiate DA binding and potency in vitro and in vivo effects of L-DOPA in a 6-OHDA contralateral turning model. In this study, we reveal that D2 PAM2 binds to a hitherto unexploited lipid-exposed extrahelical allosteric site within the D_2_R proximal to the G protein binding site. By binding to this site, it acts to potentiate the agonist efficacy by stabilizing the active state of the receptor. Molecular dynamics (MD) simulations reveal a network of residues that confer the modulator effect of PAM binding to the orthosteric site with TM5 and the tyrosine toggle switch playing the major role.

## Results

### The D2 PAM2 Displays Probe Dependence

Probe dependence, a phenomenon whereby the modulatory effect of an allosteric ligand depends on the orthosteric ligand, can provide insight into mechanisms of allosteric modulator action.^12,13^ To understand the probe dependence of the D2 PAM2, we measured its action to potentiate DA in a competition binding assay using the antagonist radioligand [^3^H]­raclopride and Flp-In-CHO cells stably expressing the human D_2_R (Figure 1). Increasing concentrations of the PAM caused a limited displacement of [^3^H]­raclopride binding and a leftward shift of the DA curve. These data could be fit to an allosteric ternary complex model to derive values of affinity of the D2 PAM2 for the unoccupied receptor (*K*_b_ = 5.5 μM), positive cooperativity with dopamine binding (α = 22, 22-fold potentiation of DA affinity), and negative cooperativity with [^3^H]­raclopride binding (α = −0.39, 2.43-fold inhibition of [^3^H]­raclopride affinity, Supporting Information S2). The inhibitory effect of D2 PAM2 on [^3^H]­raclopride binding was confirmed in a titration experiment in which increasing concentration of D2 PAM2 caused a partial displacement of [^3^H]­raclopride binding, consistent with an allosteric effect (*K*_b_ = 5.5 μM, α = 0.43, Supporting Information S3). We extended our study to an assay measuring changes in intracellular cAMP levels as a readout of G_i/o_ protein activation (Figure 1). In this assay, D2 PAM2 produced a robust potentiation of DA potency. Fitting these data to an operational model of allostery yielded values of affinity (*K*_b_ = 5.5 μM) and cooperativity with dopamine (αβ = 22, Supporting Information S2). The values of affinity determined in the functional and binding assays were not significantly different (Student’s unpaired *t*-test, *P* > 0.05). We fitted the operational model of allostery to these functional data but constrained the affinity of D2 PAM2 (*K*_b_) and its cooperativity with DA (α = 22) to those values determined in the radioligand binding experiments, allowing us to estimate the β factor that describes the modulatory action of D2 PAM2 upon DA efficacy (β = 1, Supporting Information S2). Thus, D2 PAM2 exerts little effect upon DA efficacy and appears to act as a modulator of DA affinity.

**Figure 1:**
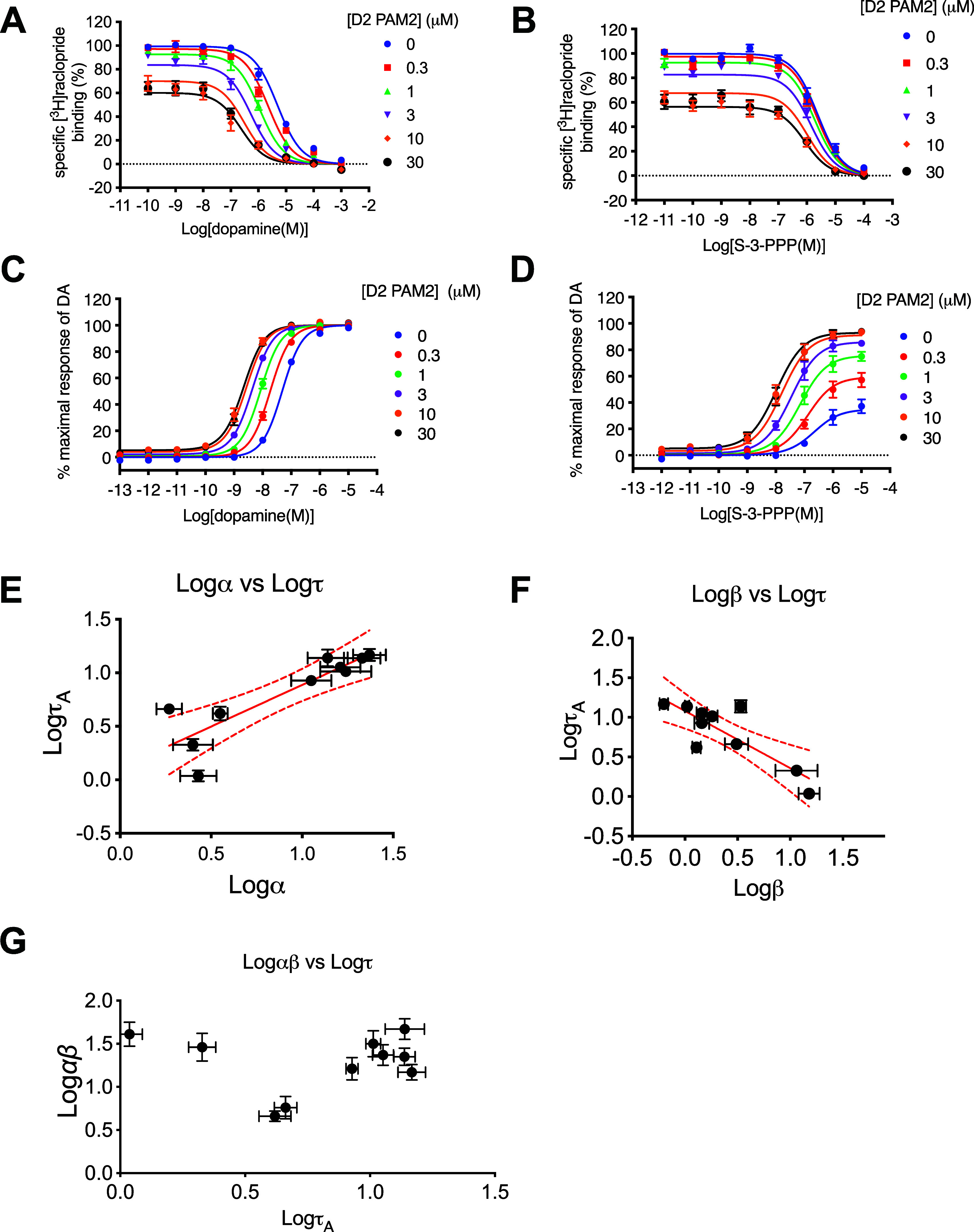
D2 PAM2 displays probe dependence. Ability of the D2 PAM2 to modulate agonist binding in a radioligand binding assay using the antagonist [^3^H]­raclopride as a tracer (A, B) in a whole cell binding assay or agonist functional activity in an assay measuring inhibition of forskolin-stimulated cAMP accumulation (C, D) of dopamine (A, C) or the partial agonist S-3PPP (B, D). Data were analyzed by an allosteric ternary complex model (binding data) or an operational model of allostery (functional data), and parameters are displayed in Supporting Information S2. We observed a positive correlation between orthosteric agonist efficacy (log τ_A_) and cooperativity between the D_2_R PAM and agonist affinity (E, log α, *r* ^2^ = 0.76) but a negative correlation between log τ_A_ and a modulatory effect on agonist efficacy (F, log β, *r* ^2^ = 0.69). No correlation was observed between orthosteric agonist efficacy (log τ_A_) and net cooperativity (G, log αβ, *r* ^2^ = 0.00). Data represent means ± S.E.M. of at least four individual experiments performed in duplicate.

We performed equivalent binding and functional studies using the partial agonist S-3PPP as the orthosteric ligand (Figure 1 and Supporting Information S2). In radioligand binding experiments, only a modest shift of the S-3PPP binding curve was observed. In the cAMP assay, increasing concentrations of the PAM acted to increase the maximal effect of S-3PPP but not its potency. Fitting these data to an allosteric ternary complex model (binding assay) and to an operational model of allostery (cAMP) revealed that although the composite modulatory effect (αβ = 29) upon S-3PPP was the same as that determined for DA, the PAM exerted a 10-fold lower cooperativity with S-3PPP affinity (α = 2.5) as compared to dopamine and an 11-fold greater modulatory effect upon S-3PPP efficacy (β = 11.5, Table 1). We extended this study to measure the modulatory action of the PAM at eight other agonists (Supporting Information S2 and Figure 1). We generated concentration–response curves for each agonist using cells that had been pretreated with or without increasing concentrations of the irreversible antagonist phenoxybenzamine. These data were fitted globally with an operational model of agonism to determine an estimate of functional affinity (*K*_A_) and efficacy (τ) for each agonist (Supporting Information S2). We observed that the greater the efficacy of the agonist, the greater the cooperativity with agonist binding (α) (Figure 1). Conversely, the lower the agonist efficacy (τ), the greater the modulatory effect on agonist efficacy (β). Thus, D2 PAM2 displays probe dependence to inhibit antagonist binding but positively modulates the binding affinity of high-efficacy agonists and the efficacy of partial agonists.

**Table 1:** Modulatory Effect of the D2 PAM2 is Not Dependent on G Proteins^e^

	+0.1 mM GppNHp	+PTX	+urea
p*K*_B_D2_ PAM^a^ (*K*_B_, mM)	4.65 ± 0.05 (45)	4.54 ± 0.03 (29)	4.53 ± 0.15 (30)
p*K*_A_DA_ ^b^ (*K*_A_, mM)	5.44 ± 0.03 (3.6)	5.37 ± 0.02 (4.3)	5.44 ± 0.02 (3.6)
Logα___[^3^H]raclo^c^ (α)	–0.19 ± 0.05 (0.65)	–0.37 ± 0.10 (0.42)	–0.06 ± 0.08 (0.87)
Logα_DA^d^ (α′)	1.30 ± 0.02 (20)	1.22 ± 0.07 (17)	1.35 ± 0.12 (22)

a The negative logarithm of the equilibrium dissociation constant of the D2 PAM2.

b The negative logarithm of the equilibrium dissociation constant of the orthosteric agonist/antagonist.

c Logarithm of the cooperativity factor between the D2 PAM2 and [^3^H]­raclopride.

d Logarithm of the net cooperativity factor between the D2 PAM2 and DA.

e Value significantly different from that obtained in the control condition, one-way ANOVA, Dunnet’s post hoc test, *P* < 0.05. The ability of the PAM to modulate the action of various agonists at the D_2L_R expressed in Flp-In-CHO cells was measured by using a cell membrane [^3^H]­raclopride binding assay. Values are expressed as means ± S.E.M. from four separate experiments.

### D2 PAM2 Acts to Increase DA Binding Affinity Independently of G Protein Binding

One explanation for this pattern of probe dependence is that D_2_R PAM2 enhances binding of G protein to the D_2_R, increasing the number of high-affinity binding sites for efficacious agonists and enhancing the efficacy of partial agonists. We performed competition binding experiments using Flp-In-CHO membranes either in the presence of 100 μM of the nucleotide (Gpp­(NH)­p), using membranes from cells pretreated with a pertussis toxin, or membranes in which urea was used to strip away G protein. In the latter two conditions, no Gα_i/o_ G protein activation was observed in a [^35^S]­GTPγS assay (Supporting Information S4). Although the affinity determined for D2 PAM2 was 4-fold lower in experiments using cell membrane preparations than that determined in the whole cell binding assays described above (*K*_B_ = 22 μM), the cooperativity with dopamine (α) was identical to that determined in the whole cell assay and the same in each condition (Table 1). These results indicate that the action of D2 PAM2 to increase DA affinity can be independent of the G protein.

In saturation binding experiments using the high-efficacy agonist [^3^H]­rotigotine and Flp-In-CHO D_2_R cell membranes, the presence of the 10 μM D2 PAM2 (Figure 2 and Supporting Information S2) did not change [^3^H]­rotigotine affinity in the absence of nucleotides. In the presence of 100 μM Gpp­(NH)­p, the affinity of [^3^H]­rotigotine was 9-fold lower, and the addition of the D2 PAM2 caused a 14-fold increase in affinity.

**Figure 2:**
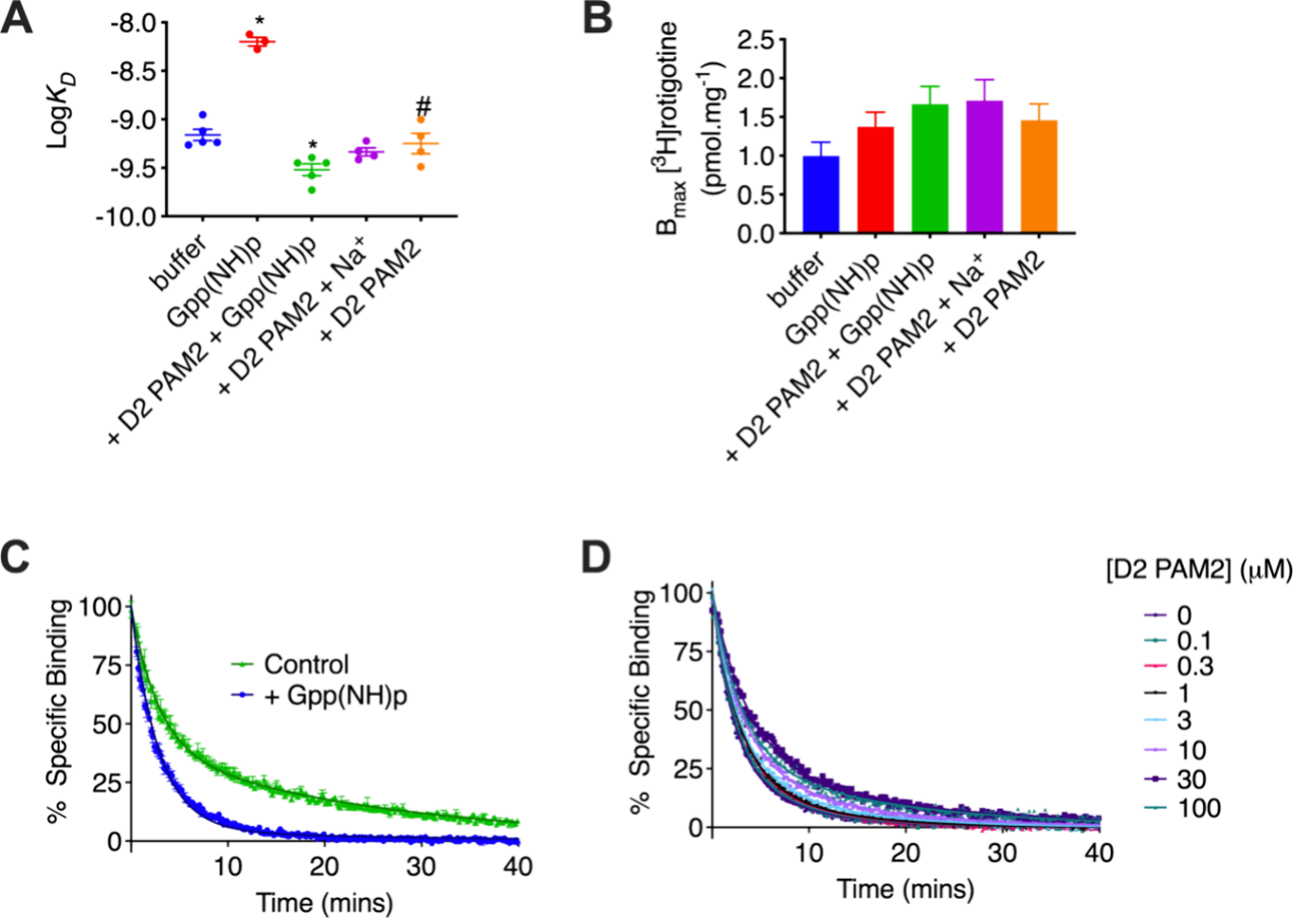
D2 PAM2 increases the slow phase of agonist dissociation in the absence of G protein coupling. Saturation binding studies using the radiolabeled agonist [^3^H]­rotigotine and membranes of Flp-In-CHO cells stably expressing the D_2L_R revealed that (A) D2 PAM2 caused a significant increase in [^3^H]­rotigotine affinity in the absence or presence of 100 μM Gpp­(NH)p but (B) no significant change in the number of [^3^H]­rotigotine binding sites. Data represent means ± standard error of at least four individual experiments performed in duplicate. * = significant difference to buffer conditions, # = significant difference to Gpp­(NH)p conditions. One-way ANOVA, Tukey’s post hoc test, *P* <0.01 Tag-lite TR-FRET binding experiments using membranes of HEK293 cells stably expressing terbium-labeled SNAP-D_2S_R, and the fluorescent agonist PPHT-d1 revealed that (C) 100 μM Gpp­(NH)p decreased the percentage of slow-phase agonist dissociation. In the presence of 100 μM Gpp­(NH)­p, the D2 PAM2 increased slow-phase dissociation (D). Data represent means ± S.E.M. of at least four individual experiments performed in duplicate.

The dissociation of a radiolabeled agonist from the D_2_R has been shown to display slow and fast phases. Adding a high concentration of GTP to uncouple the D_2_R from G_i/o_ G proteins caused a decrease in the slow phase of dissociation.^14^ We used a TR-FRET binding assay that measured the binding of a fluorescently labeled D_2_R agonist (PTHP-d1) to a SNAP-tagged terbium-labeled D_2_R receptor (Figure 2 and Supporting Information S5.^15,16^ PTHP-d1 dissociation was initiated with a high (isotopic) concentration of 30 μM spiperone with or without the presence of D2 PAM2. We found that the addition of Gpp­(NH)p to ablate receptor-G protein coupling caused a decrease in the percentage of slow-phase agonist dissociation (Figure 2 and Supporting Information S6). In the presence of Gpp­(NH)­p, however, increasing concentrations of the PAM caused a concentration-dependent increase in the slow phase of dissociation, consistent with the PAM acting in a manner akin to G protein by stabilizing a conformation of the receptor from which the agonist dissociates slowly. Thus, the action of the PAM to enhance the agonist affinity appears to be similar to, but not dependent on, the action of G proteins.

We then extended our analysis to investigate whether D2 PAM2 displayed differential modulatory action across different signaling pathways and regulatory end points. We measured the action of the D2 PAM2 to modulate the action of DA in assays measuring ERK1/2 phosphorylation, β-arrestin recruitment, and receptor internalization (Supporting Information S7 and S8). In each assay, D2 PAM2 modulated the action of dopamine with no significant difference between the size of the overall modulatory effect of the D2 PAM2 (αβ) across the different assays. Interestingly, in the case of arrestin recruitment, we observe that D2 PAM2 acts to modulate both dopamine potency and efficacy, with the latter indicated by an increase in the *E*_max_ of dopamine. This suggests that DA gives a partial response with respect to the maximal possible level of arrestin recruitment to the D2R. In this respect, the action of the D2 PAM2 on DA in this assay is akin to the modulatory effect on partial agonist efficacy observed in the cAMP assay. The intrinsic efficacy (τ_B_) of D2 PAM2 in the different assays correlates with the potency of DA, suggesting that the level of allosteric intrinsic efficacy observed is determined by the signal amplification of the particular signaling end point being measured.

### D2 PAM2 Displays Subtype Selectivity at the D_2_-like DRs

The orthosteric DA binding site is conserved across the DRs.^17–20^ Allosteric sites are less likely to be conserved, and D2 PAM1 does not display activity at D_1_Rs with D2 PAM2 proposed to display a similar selectivity.^11^ To test whether the D2 PAM2 might display subtype selectivity across the D2-like DRs, we investigated the ability of the PAM to potentiate binding at the D_3_R and D_4_R using [^3^H]­raclopride (or [^3^H]­spiperone for D_4_R) competition binding assays (Figure 3). The affinity of D2 PAM2 and its cooperativity with DA were not significantly different at the D_3_R as compared to the D_2_R (Supporting Information S9). In contrast, no activity was observed at the D_4_R (Figure 3), suggesting that either D2 PAM2 does not bind the D_4_R or it binds but does not modulate DA binding.

**Figure 3:**
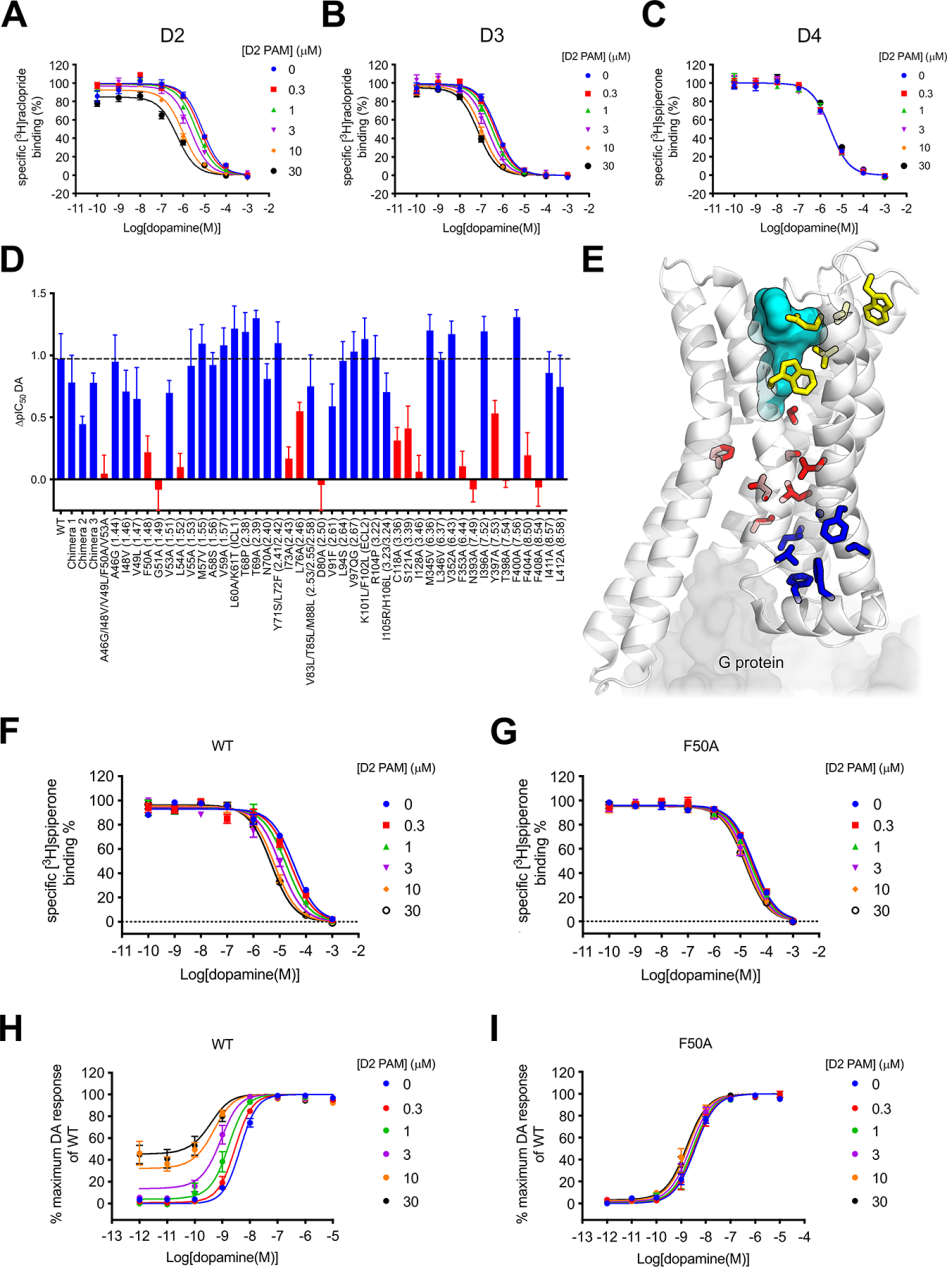
Mutation of residues within an intracellular pocket of the D_2_R abrogates the action of D2 PAM2. (A) D2 PAM2 displays subtype selectivity. We measured the ability of the D2 PAM2 to modulate the action of DA in radioligand binding assays using [^3^H]­raclopride as a tracer for D_2_R (A) and D_3_R (B) and [^3^H]­spiperone as a tracer for D_4_R (C). Experiments were conducted using membrane preparations of Flp-In-CHO cells expressing each receptor. (D) Ability of 10 μM D_2_R PAM to cause an increase in the potency (pIC_50_) of DA at various chimeric D_2_/D_4_ or mutant D_2_ receptors. Those chimeras/mutants with a significant decrease in fold shift are highlighted in red (one-way ANOVA, Dunnets’s post hoc test, *P* <0.01). (E) Point mutations listed in Tables 2 and 3 and Supporting Information S13 shown on an experimentally determined structure of the D_2_R (PDB accession code 6VMS). The result of a residue mutation indicated by color-coding: blue, inhibition of the D2 PAM2 effect, residue within the putative allosteric pocket; red, inhibition of the D2 PAM2 effect, residue involved in GPCR activation; yellow, no effect on D2 PAM2 action. The gray surface represents the G protein, and the cyan surface depicts the location of the orthosteric binding pocket. (F–I) Ability of D2 PAM2 to potentiate DA binding in a [^3^H]­raclopride binding assay (F, G) or the functional action of DA (H, I) at the WT or at the TM1 mutant F50^1.48^A. Data represent means ± S.E.M. of four individual experiments performed in duplicate.

### Site-Directed Mutagenesis Indicates that D2 PAM2 Binds to a Membrane-Exposed Extrahelical Pocket

The D_2_R shares greater identity to the D_3_R (75% within the TM domains) as compared to the D_4_R (50% within the TM domains). We used the subtype selectivity displayed by D2 PAM2 to identify its binding site. We first generated three chimeric D_2/4_Rs, one that includes D_2_R residues up until the top of TM3 (Chimera 1), one up until the start of TM6 (Chimera 2), and one up until the end of TM7 (Chimera 3). In a [^3^H]­spiperone competition binding assay to measure the ability of a 10 μM concentration of D2 PAM2 to increase the affinity of DA, D2 PAM2 displayed activity at all chimeric receptors (Figure 3D). This suggested that the residues that differ between the D_2_R and the D_4_R that are important for the binding and effect of D2 PAM2 lie within the first two TM domains, the N-terminus or ICL1. We mutated these D_2_R residues to their D_4_R equivalents and, using a [^3^H]­spiperone competition binding assay, assessed the ability of 10 μM D2 PAM2 to potentiate DA binding at each mutant as indicated by the fold increase in the IC_50_ of DA. This approach revealed that mutation of five TM1 residues to equivalent D_4_R residues caused a loss of the effect of D2 PAM2 (A46^1.44^G/I48^1.46^V/V49^1.47^L/F50^1.48^A/V53^1.53^A, Figure 3D). We then investigated the effect of mutation of each of these residues separately and found that the mutation F50^1.48^A caused a significant loss in the effect of the PAM. A [^3^H]­spiperone competition binding interaction experiment revealed that this mutation (p*K*_B_ = 5.57 ± 0.25, 3 μM; log α = 0.33 ± 0.03, α = 2.1) caused no significant change in D2 PAM2 affinity but a 5-fold decrease in cooperativity with DA as compared to WT (p*K*_B_ = 5.04 ± 0.17, 9 μM; log α = 1.05 ± 0.10, α = 11) (Figure 3E–H). This residue is lipid-exposed and oriented away from the receptor core, close to residues in helix 8 (Figure 3E). We extended our mutagenesis analysis to nearby residues in TM1, TM2, TM7, and helix 8. These residues were conserved between the D_2_R, the D_3_R, and the D_4_R; so, we mutated each residue to alanine. In TM1, the mutations G51^1.49^A and L54^1.52^A, proximal to and a turn down from F50^1.48^, respectively, completely abrogated the effect of D2 PAM2. I73^2.43^A and L76^2.46^A caused a decrease in the IC_50_ shift caused by D2 PAM2 (Figure 3D, Table 2, and Supporting Information S10). At the bottom of TM7, the mutations Y397^7.53^A and T398^7.54^A caused a decrease and a complete loss of the D2 PAM2 effect, respectively (Figure 3D, Table 2, and Supporting Information S10). Finally, F404^8.50^A and F408^8.54^A within helix 8 caused a complete loss of the D2 PAM2 effect (Figure 3D, Table 2, and Supporting Information S10). Of note, the mutations (F50^1.48^A, G51^1.49^A, L54^1.52^A, I73^2.43^A, F404^8.50^A, and F408^8.54^A) that decreased/abrogated the effect of D2 PAM2 did not change the affinity of DA (Table 2 and Supporting Information S10), indicating that these mutations have not caused a conformational change to the D_2_R that impacts DA binding.

**Table 2:** Effect of D2 PAM2 Inhibited by the Mutation of Residues within an Intracellular Binding Pocket^g^

	DA			D2 PAM2		
	p*K*_i_ ^a^ (*K*_i_, μM)	pEC_50_ ^b^ (EC_50_, nM)	ΔpIC_50_ + 10 μM PAM (fold shift)	p*K*_B_ ^c^ (*K*_B_, nM)	log αβ^d^ (αβ)	log τ^e^ (τ)
WT	4.46 ± 0.17 (35)	8.35 ± 0.05 (4)	0.97 ± 0.20 (9)	5.02 ± 0.14 (9.5)	1.25 ± 0.14 (18)	0.06 ± 0.05 (1)
F50^1.48^A	4.54 ± 0.08 (29)	8.43 ± 0.04 (4)	0.22 ± 0.12^f^ (1.7)	5.32 ± 0.28 (5)	0.39 ± 0.07 (2.5)^f^	–1.35 ± 0.22 (0.04)
G51^1.49^A	4.45 ± 0.10 (35)	8.25 ± 0.05 (6)	–0.08 ± 0.17^f^ (0.8)			
N52^1.50^A				4.89 ± 0.21 (12.8)	1.18 ± 0.28 (15)	0.23 ± 0.07 (1.69)
L54^1.52^A	4.80 ± 0.07 (16)	8.24 ± 0.04 (6)	0.10 ± 0.11^f^ (1.3)			
I73^2.43^A	4.86 ± 0.09 (14)	8.48 ± 0.04 (3)	0.17 ± 0.09^f^ (1.5)	5.24 ± 0.16 (6)	0.79 ± 0.07 (6.1)^f^	–1.11 ± 0.16 (0.08)
T398^7.54^A	4.09 ± 0.06 (81)	7.00 ± 0.03^f^ (100)	–0.01 ± 0.05^f^ (1)	5.35 ± 0.17 (4)	0.46 ± 0.05 (2.9)^f^	–1.40 ± 0.22 (0.04)
F404^8.50^A	5.36 ± 0.09 (4)	7.26 ± 0.06^f^ (550)	0.19 ± 0.18^f^ (1.5)			
F408^8.54^A	4.79 ± 0.08 (16)	7.38 ± 0.07^f^ (42)	–0.07 ± 0.14^f^ (0.85)			

a The negative logarithm of the equilibrium dissociation constant of DA in the radioligand binding assay.

b The potency of DA in the functional assay.

c The negative logarithm of the equilibrium dissociation constant of the D2 PAM2 determined in the functional assays.

d Logarithm of the net cooperativity factor between the D2 PAM2 and DA determined in the functional assay.

e Estimate of the logarithm of the modulatory factor and orthosteric ligand efficacy determined in the functional assay.

f Value significantly different from that obtained in the control condition, one-way ANOVA, Dunnet’s post hoc test, *P* < 0.05.

g The affinity of DA and the fold shift in DA IC_50_ caused by 10 μM PAM at the WT or mutant SNAP-D_2S_R expressed in Flp-In-CHO cells were measured using a cell membrane [^3^H]­spiperone assay. The affinity of the D2 PAM2, its intrinsic efficacy, and cooperativity with DA at WT or mutant SNAP-D_2S_R expressed in Flp-In-CHO cells were measured in a functional cAMP assay using a BRET biosensor.

The mutations G51^1.49^A and L54^1.52^A that caused the loss of the D2 PAM2 effect in the binding assay also abrogated its action in a cAMP inhibition assay (Table 2 and Supporting Information S10). F50^1.48^A caused a 7-fold decrease in the cooperativity with DA. I73^2.43^A caused 2.8-fold cooperativity with DA. DA displayed a similar potency at all four mutants, and they displayed cell surface expression similar to WT (Supporting Information S11), indicating that the effect of these mutations is restricted to the activity of D2 PAM2 rather than the ability of the D_2_R to bind DA and activate G protein. T398^7.54^A also caused a 6-fold decrease in cooperativity with DA with no change in the affinity of D2 PAM2. This mutation also caused a 23-fold decrease in the potency of DA, suggesting that the mutation of this residue also decreases the efficiency with which DA activates the D_2_R. Finally, F404^8.50^A and F408^8.54^A caused a complete loss of the D_2_R PAM effect and 12-fold and 9-fold decreases in DA potency (Table 2 and Supporting Information S10). This suggests that these helix 8 residues also contribute to D_2_R Gα_i/o_ G protein coupling. In contrast, the mutation of residues previously proposed to form an extracellular binding pocket of D2 PAM2 (V91^2.61^A, L94^2.64^A, E95^2.65^A, W100^ECL1^A, I184^ECL2^A, and W384^7.40^A) had no significant effect on the affinity of D2 PAM2 or its cooperativity with DA (Figure 3D and Supporting Information S12 and S13).^21^ These experimental data have identified residues from TMs 1, 2, and 7 and helix 8 that decrease or abrogate the effect of D2 PAM2 (Figure 3). While such mutations may impact D2 PAM2 function indirectly, the selective effect of some mutations on D2 PAM2 without changing DA affinity or potency provides evidence that these residues might form the PAM binding pocket.

Several D_2_R structures were recently determined by crystallography and cryo-EM, including inactive- or active-state conformations.^18,22–27^ None of these structures contain a bound allosteric modulator, but the structures can be used to identify potential pockets with the aid of the mutagenesis data and clarify what are likely to be D2 PAM2 binding site residues versus those that might indirectly contribute to its allosteric effect. Six of the point mutations relevant for D2 PAM2 activity (F1.48, G1.49, L1.52, I7.51, T7.54, and F8.54) are located between lipid-exposed intracellular parts of TM1, TM7, and H8 (Figure 3E). Visual inspection of available cryo-EM structures reveals that these residues, together with residues V1.45, V1.53, P7.50, and T7.55, form a cleft on a receptor transmembrane surface. The bottom of the pocket is formed by G1.49, P7.50, and V1.53, while V1.45, F1.48, I7.51, and F8.54 form a ridge around it. The area abuts several conserved residues that play a role in the class A receptor activation mechanism, such as the ^7.49^NPxxY^7.53^ motif and the most conserved residues of TM1 (N1.50) and H8 (F8.50). P7.50 introduces a kink to TM7, which exposes the backbone carbonyl of the A7.47 residue to the hydrophobic environment of the lipid membrane. Notably, this carbonyl oxygen forms a hydrogen bond with a water molecule in one D_2_R crystal structure (PDB accession code 6CM4). Thus, we provide evidence that D2 PAM2 binds to a lipid-exposed extrahelical pocket defined by residues from TMs 1 and 7 and helix 8. Interestingly, the predicted allosteric pocket is also close to a palmitoylation site in H8, which has been demonstrated to play an important role in receptor function^28^ and could contribute to pocket formation.

As D2 PAM2 shows no activity at D_4_R, comparison of the experimentally determined D_2_R and D_4_R structures could hold important information about the allosteric binding site (Supporting Information S14). Surface representations of the receptor subtypes reveal a relatively deep cleft in the D_2_R, whereas the corresponding cavity in D_4_R is wider and shallower (Supporting Information S14). Whereas bulky residues contribute to formation of the allosteric pocket in D_2_R (i.e., V1.45, F1.48, and I7.51), the corresponding side chains in the D_4_R are smaller (A1.45, A1.48, and V7.51, respectively). These differences together affect the overall shape of the homologous cleft in D_4_R, which provides an explanation for D2 PAM2 subtype specificity.

### Prediction of PAM Binding Mode Using Enhanced Sampling Simulations

The mutagenesis studies identified a possible allosteric site locations and enabled enhanced sampling MD simulations to predict the D2 PAM2 binding mode in this site. In our calculations, we restrained the ligand within the area of interest and explored possible protein-modulator binding conformations. Of the available experimental structures of D_2_R, one was solved in a membrane environment with a complete C-terminus (PDB ID 6VMS) and hence provided the best description of the predicted extrahelical pocket. Therefore, this structure was selected for our simulations. The simulations were performed using an all-atom force field, providing a detailed and accurate representation of the interactions between the allosteric modulator and the receptor, lipid membrane, and water molecules.

We combined walls of potential with well-tempered metadynamics to constrain D2 PAM2 in the identified pocket and exhaustively sample relevant binding states. The sampling was restricted within a sphere of a 5 Å radius, defined as a distance between the ligand’s center of mass and the center of the allosteric pocket between TM1, TM7, and H8. The well-tempered metadynamics method is an MD technique that enhances sampling of relevant states. Metadynamics uses Gaussian-shaped artificial potentials that encourage a system of interest to cross energy barriers and visit sparsely populated states. In well-tempered metadynamics, the height of these potentials is adaptive so that consecutive potentials applied to a particular state are weaker, which leads to smooth convergence. The multiple-walker metadynamics simulation was conducted with five walkers. Convergence was achieved after ∼380 ns per walker (Supporting Information S15), and the resulting free energy surface (FES) allowed comparisons between possible binding modes. As both D2 PAM1 and D2 PAM2 share the same scaffold and, therefore, are likely to bind to the same pocket, we applied the same simulation protocol to both compounds. As shown in Figure 4B, both D2 PAM1 and D2 PAM2 are characterized by similar free energy landscapes, with energy minima falling in the same landscape area. This indicates that the ligands are predicted to form similar complexes. The landscapes reveal two energy wells, of which one is deeper and hosts the global free energy minimum of the modulator-receptor complex. The simulation frames corresponding to the low energy wells were extracted and clustered for the D2 PAM2. Central frames of the largest clusters were further evaluated by performing short MD simulations. In agreement with the free energy landscapes, the binding mode from the global free energy minimum showed the highest stability in the simulations and was further analyzed (Figure 4A).

**Figure 4:**
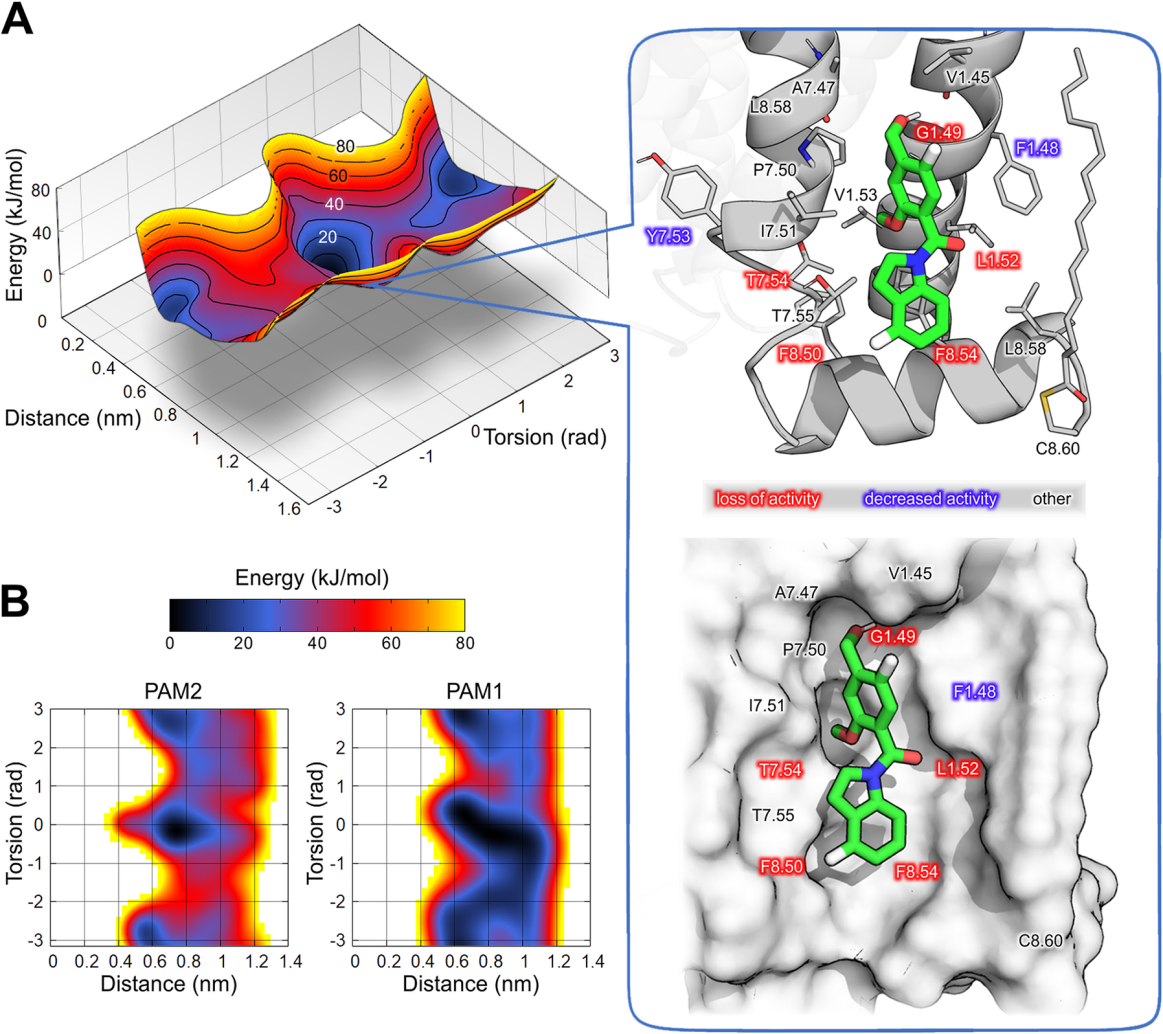
Enhanced sampling MD simulations reveal the preferred binding mode of the D2 PAM2. (A) Free energy landscape of modulator translation and rotation reveals clearly defined minima (left panel). A simulation snapshot corresponding to the global minimum shows how PAM2 fills the extrahelical cavity and interacts with residues pinpointed by site-directed mutagenesis (right panel; D2 PAM2 shown as green licorice; receptor residues depicted with a stick or surface representation in the upper and lower frame, respectively). (B) Free energy landscapes obtained for D2 PAM2 and D2 PAM1, suggesting a similar binding mode of both modulators.

In the predicted binding mode, D2 PAM2 fills a deep cavity where V1.45, A7.47, I7.51, and F1.48 residues surround the hydroxymethylphenyl moiety. The hydroxyl group is buried between TM1 and TM7 near the conserved P7.50 residue and displaces the water molecule observed in the D_2_R crystal structure (Supporting Information S6). A π–π stacking interaction is observed between the indole moiety of D2 PAM2 and the F8.54 side chain. The C-terminal palmitoylated cysteine additionally surrounds the binding area. The binding mode agrees with the site-directed mutagenesis experiments and provides insight into the role of the surrounding residues. The G1.49A mutation introduces a methyl group into the cavity of the binding site that anchors the ligand, decreasing the depth of the pocket. The relatively bulky L1.52 and F1.48 side chains surround the allosteric pocket, and therefore, L1.52A and F1.48A result in a more exposed cavity. Mutation of any of the phenylalanine residues located at H8 leads to a loss of favorable π–π stacking interactions with the indole moiety of the ligand. The I2.43, L2.46, Y7.53, and T7.54 residues, which also affected D2 PAM2 activity in the mutagenesis studies, do not directly interact with the modulator in the predicted binding mode (Figure 4). However, most of these residues are conserved and known to constitute molecular switches in class A GPCRs (e.g., the NPxxY motif).^29^ Thus, these mutations are unlikely to interfere in modulator binding but rather the cooperative communication between orthosteric and allosteric sites.

### D2 PAM2 Stabilizes the D_2_R Active State

To validate the binding pose further and understand the mechanism of allosteric modulation, we conducted a series of all-atom unbiased MD simulations. We prepared simulations with D_2_R in the active state, in the presence or absence of the α subunit of G_i_ protein. The active-state receptor structure in the absence of G protein was used to prepare complexes with D2 PAM2, the agonist rotigotine, or both of these ligands. All simulations were run in six replicas, resulting in a total of 6 μs of production run per complex. In all simulations, the ligand poses were maintained and the receptor-Gα complexes were stable. The stability of the receptor-modulator complex and interactions contributing to binding were analyzed by identifying the different types of interactions formed (Supporting Information S17 and S18). The interaction barcodes revealed that the hydrogen bonding of the modulator’s hydroxyl group with A7.47 is one of the most stable interactions, along with hydrophobic contacts with I7.51 and F8.54.

The set of simulations was designed to assess the influence of ligand configurations on receptor conformation. In the absence of any small-molecule ligands or the intracellular coupling partner, the receptor would be expected to relax toward an inactive conformation. Meanwhile, the presence of either Gα, rotigotine, or D2 PAM2 should stabilize the active state. Such effects should be possible to detect in MD simulations, given the correct input and sufficient timescale.^30^ Heavy-atom root-mean-square deviation (RMSD) calculations showed that the drift is most pronounced if the G protein or an agonist is absent (Supporting Information S19). Root-mean-square fluctuation (RMSF) analysis pointed to ICL3 and TM6 as the main sources of the drift (Supporting Information S20). Interestingly, while the combination of D2 PAM2 and rotigotine seems to stabilize ICL3, it has the opposite effect on ICL2, which is suggested to play an important role in GPCR activation.^31^

To identify, quantify, and assess conformational changes occurring in the simulations, principal component analysis (PCA) was employed. This method identifies the most pronounced variance in the system. When the method is applied to trajectories, it helps to sift relevant protein motions induced by the presence or absence of ligands. All PCA calculations were performed on concatenated and fitted trajectories. First, we assessed if MD simulations were able to distinguish between conformations of liganded and unliganded receptors by performing Cartesian PCA (cPCA) on a conformational ensemble from simulations of the apo receptor and receptor bound to both rotigotine and D2 PAM2. The agonist- and PAM2-bound receptor complex maintained more active-like conformations within the analyzed conformational space, as described by the first principal component (PC1) (Figure 5A). The conformational changes involved in deactivation of the apo receptor were clearly caught in the simulations, with the broadly accepted hallmark of deactivation—an inward shift of TM6—heavily pronounced in the final frames of the apo simulations. Then, cPCA was performed on an ensemble containing trajectories that included only one of the binders at the same time: Gα, rotigotine, or D2 PAM2 (Figure 5B) to compare their effects separately. The analysis showed that the second principal component (PC2) separates conformational states of the apo receptor, which is characterized by PC2 values below zero, from the Gα-bound receptor or D2 PAM2-bound receptor, both of which have PC2 values around or above zero. Thus, the PCAs allow sorting of trajectories depending on the presence or absence of factors stabilizing the active state. The results indicate that D2 PAM2 stabilizes a receptor conformation similar to the Gα-bound receptor and that rotigotine and PAM2 are able to stabilize active-like states over the course of a 1 μs simulation.

**Figure 5:**
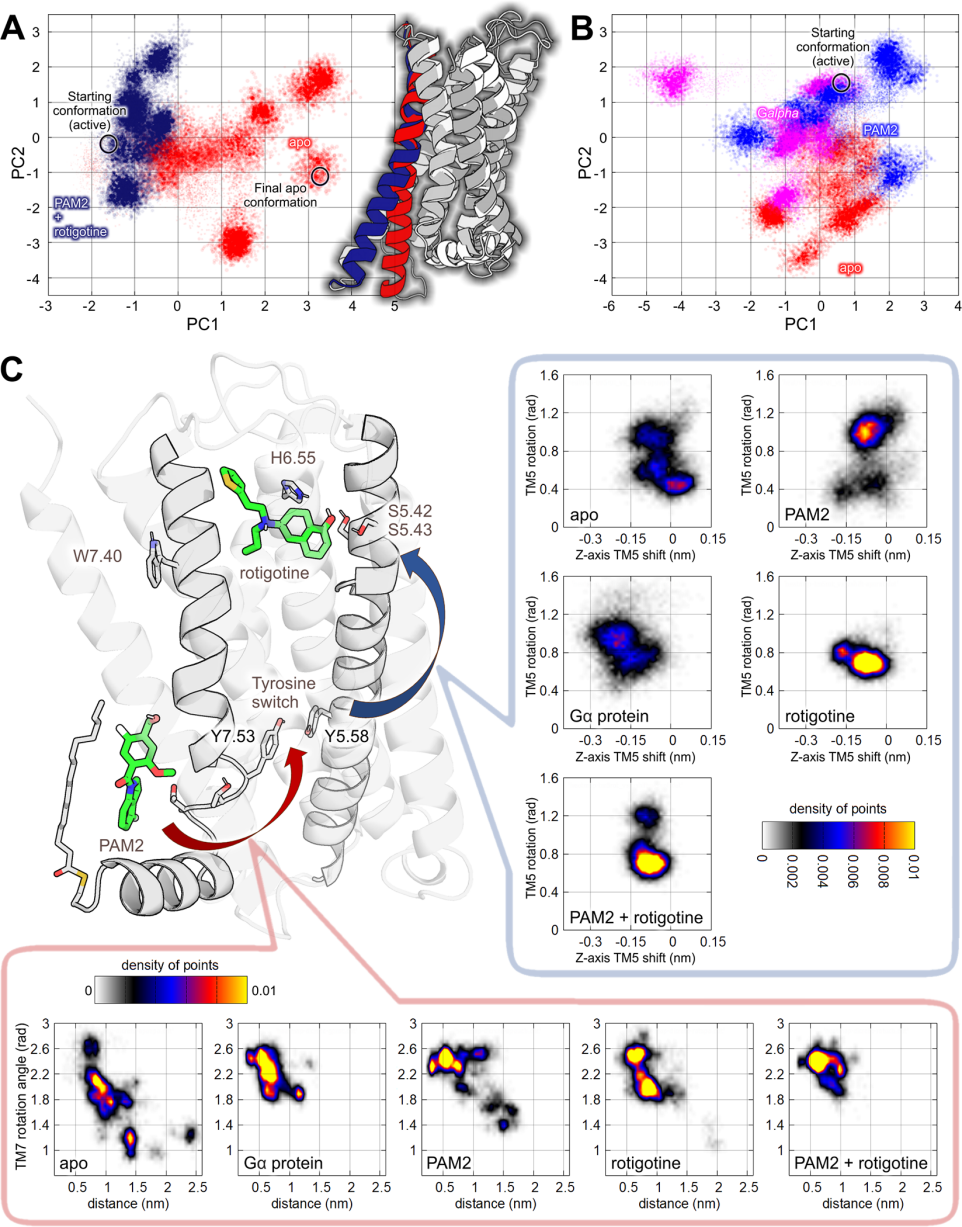
Analysis of the unbiased MD simulations. (A) Cartesian principal component analysis (cPCA) of concatenated trajectories, including apo and all-holo receptors (PAM and rotigotine). PCA was run on an atom selection including both orthosteric and allosteric sites, as well as conserved GPCR molecular switches. Color-coded cartoon representations of the corresponding receptor conformations are presented on the right. (B) cPCA on trajectories of the receptor bound to either PAM, Gα protein, or in the apo state. (C) Protein motions related to D2 PAM2 binding. Right panel: TM5 rotation [an angle defined by Cβ of S5.42, center of geometry (COG) of TM5, and COG of TM2 at the height of the serine] against the TM5 shift in the *Z*-axis, perpendicular to the membrane (the distance between a center of geometry of helices TM2, TM3, TM4, and TM7 and a center of geometry of TM5); lower panel: distribution of TM7 rotation values (measured at the NPxxY motif) against the tyrosine switch conformation (the distance between hydroxyl groups of Y7.53 and Y5.58).

Next, trajectories were filtered to include only the motions described by the most relevant principal components, with the goal to identify the conformational changes involved. Detailed analysis revealed a series of correlated motions participating in propagation of conformational changes between the allosteric site, G protein binding interface, and the orthosteric pocket (Figure 5C). The conserved tyrosine switch, composed of residues Y7.53 and Y5.58, appears to play a central role in the allosteric mechanism (Figure 5C, lower panel). Furthermore, the two-way communication between the tyrosine switch residues and the orthosteric pocket seems to be governed by rotation and a vertical shift of TM5 (Figure 5C, right panel). Rotation of TM5 and its motions along the *Z*-axis affect both tyrosine switch formation at the G protein interfaces and arrangement of S5.42 and S5.43 residues in the orthosteric binding pocket, which play an important role in orthosteric ligand binding.

The distribution of values describing TM7 rotation against the tyrosine switch conformation shows a clear tendency (Figure 5C, lower panel). In the apo state, the most populated area of the tyrosine switch distance (measured between the hydroxyl oxygens) ranges between 6 and 15 Å. The presence of any ligands (Gα protein, rotigotine, D2 PAM2, or the D2 PAM2-rotigotine combination) shifts the most populated range toward shorter distances and narrows the visited range. This clearly shows that in the absence of any ligands, the tyrosines drift apart due to the rotation of the conserved ^7.49^NPxxY^7.53^ motif. In contrast, the presence of either D2 PAM2 in the allosteric pocket, Gα protein at the intracellular interface, or rotigotine at the orthosteric pocket stabilizes TM7 in a conformation favoring the Y5.58–Y7.53 interaction. Therefore, these ligands appear to stabilize the tyrosine switch in the active-state-like conformation.

The calculated densities of receptor states in terms of the TM5 conformation show that in the apo state, this helix rotates within a range of nearly 1 radian (Figure 5C, right panel). It moves unrestricted along the *Z*-axis within a range of nearly 3 Å (a majority of the values span between −1.5 and 1 Å), with the weakly pronounced most frequently visited state at the angle of 0.4 rad and a shift around 0 Å. The presence of Gα protein shifts these values to 1 rad and −2 Å, respectively. D2 PAM2 mimics this effect and stabilizes the receptor conformation, revealing a clear maximum of density of states at 1 rad and around −1 Å, respectively. This resembles the shift induced by rotigotine, suggesting that D2 PAM2 stabilizes TM5 conformations favorable for full agonist binding.

The data presented in Figure 5 are in agreement with the in vitro results of the TR-FRET measurements of F-PTHP binding as well as with competition binding experiments on pretreated Flp-In-CHO membranes, described above. The MD results support the hypothesis that D2 PAM2 acts similarly to G proteins but does not depend on their presence. The simulations provide a plausible explanation of how D2 PAM2 stabilizes the active state of the receptor, pinpointing the tyrosine switch and TM5 as central to signal transmission. The stabilized state is characterized by the orthosteric site conformation favorable for full agonist binding, thus explaining the increase in the slow dissociation phase upon D2 PAM2 presence.

### Mutations of Residues Important for the Isomerization of GPCRs to an Active State Modulate the Action of D2 PAM2

We provide evidence that D2 PAM2 binds to an extrahelical pocket to modulate the binding of DA in a manner similar to that of G protein binding. Thus, residues important for communication between the orthosteric agonist binding site and the G protein binding site are likely important for the modulatory effect of D2 PAM2. The D2 PAM2 binding pocket lies close to the NPxxY motif.^32^ The mutation of N393^7.49^A of the NPxxY motif caused a large decrease in DA potency and abrogated the effect of D2 PAM2 (Table 3 and Supporting Information S16). The mutations Y397^7.53^A and T398^7.54^A caused a decrease in the cooperativity between the D2 PAM2 and DA (Table 3 and Supporting Information S16), supporting the observations derived from MD simulations. N7.49 forms a hydrogen bond network with D2.50 and F6.44.^33^ D2.50 forms the conserved Na^+^ binding pocket along with Ser3.39.^34,35^ DA displays a 2200-fold lower potency at S121^3.39^A. As S121^3.39^A only shows 20-fold lower affinity for DA, the efficacy of DA at this mutant must be reduced. While the ability of the D2 PAM2 to potentiate DA binding was compromised at this mutant, the functional data revealed that the D2 PAM2 was bound with WT affinity and modulatory effects (Table 3 and Supporting Information 8). The mutation L76^2.46^A, a residue that is part of a network that supports Na^+^ binding, caused a 5-fold decrease in the cooperativity between the D2 PAM2 and DA (Table 3 and Supporting Information S16). At D80^2.50^A, the D2 PAM2 could not potentiate DA binding but increased both the potency and maximal effect of DA in a functional assay with an affinity and cooperativity not different from that at the WT D_2_R (Figure 6). This mutation caused a 10,000-fold decrease in DA potency such that it equals the functional affinity of DA (pEC_50__DA = 4.48 ± 0.11, p*K*_A__DA = 4.51 ± 0.17; Table 3 and Figure 6). The cell surface expression of this mutant is not significantly different from that of the WT; thus, we conclude that this mutation decreases the efficacy of DA (Table 3). A similar pattern was observed on mutation of other residues highlighted as important for the isomerization of class A GPCRs into the active G protein-coupled state on agonist binding including F^6.44^A (part of the conserved P^3.40^I^5.50^F^6.44^ motif), I128^3.46^A, and C118^3.36^ (which lies one turn down from D114^3.32^ that forms a salt bridge with the positively charged aliphatic amine of most orthosteric D_2_R ligands). F353^6.44^A, I128^3.46^A, and C118^3.36^A caused a decrease in DA potency that cannot be attributed solely to a loss of DA affinity, indicating that these residues cause a decrease in DA efficacy. D2 PAM2 is unable to potentiate DA binding at these transmission network mutants, but our functional experiments reveal that D2 PAM2 binds these mutants with WT affinity and acts to increase the maximal effect (efficacy) of DA, analogous to the effect of D2 PAM2 on partial agonists at the WT D_2_R.

**Figure 6:**
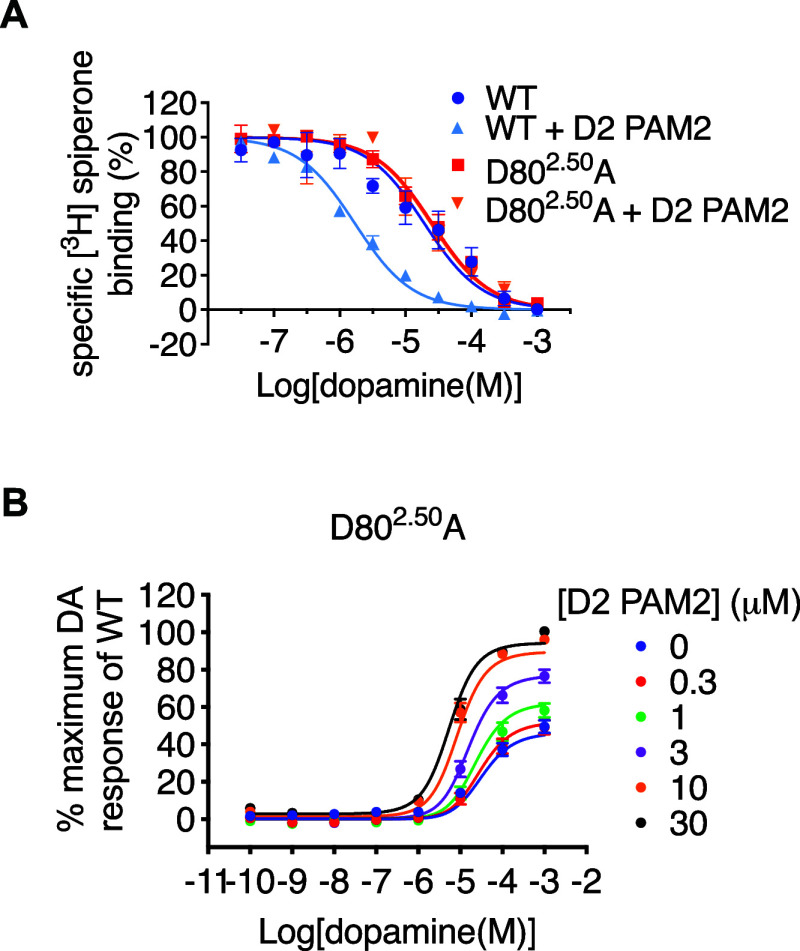
A residue that controls the isomerization of the D_2_R into an active state also controls the modulatory effect of the D2 PAM2 on DA binding. (A) In a [^3^H]­spiperone binding assay, 10 μM D2 PAM2 can potentiate the binding of DA at the D_2_R but not at the D80^2.50^A mutant. DA retains the WT affinity at the D80^2.50^A mutant. (B) In a functional cAMP assay, DA displays a reduced pEC_50_ and maximal effect at the D80^2.50^A mutant as compared to the WT D_2_R. The D_2_R PAM binds the D80^2.50^A mutant with WT affinity and acts to increase DA potency and efficacy. Data represent means ± S.E.M of four individual experiments performed in duplicate.

**Table 3:** Effect of D2 PAM2 on DA Binding Inhibited by Mutation of Residues Involved in Receptor Activation^g^

	DA			D2 PAM2		
	p*K*_i_ ^a^ (*K*_i_, μM)	pEC_50_ ^b^ (EC_50_, nM)	ΔpIC_50_ + 10 μM PAM (fold shift)	p*K*_B_ ^c^ (*K*_B_, μM)	log αβ^d^ (αβ)	log τ^e^ (τ)
WT	4.46 ± 0.17 (35)	8.35 ± 0.05 (4)	0.97 ± 0.20 (9)	5.02 ± 0.14 (9.5)	1.25 ± 0.14 (18)	0.06 ± 0.05 (1)
L76^2.46^A	4.33 ± 0.05 (47)	7.84 ± 0.07 (14)	0.55 ± 0.07 (3.5)^f^	5.52 ± 0.31 (3)	0.56 ± 0.09 (3.6)	–3
D80^2.50^A	4.46 ± 0.17 (35)	4.48 ± 0.11 (33,110)	–0.04 ± 0.27 (0.9)^f^	4.82 ± 0.18 (1.5)	0.96 ± 0.25 (9.0)	–0.78 ± 0.25 (0.17)
C118^3.36^A	3.92 ± 0.10 (120)	5.91 ± 0.04 (1230)	0.31 ± 0.10 (2)^f^	4.82 ± 0.15 (15)	1.30 ± 0.13 (20)	–0.24 ± 0.05 (0.57)
S121^3.39^A	3.22 ± 0.10 (603)	5.01 ± 0.03 (9772)	0.41 ± 0.18 (2.6)^f^	4.83 ± 0.11 (15)	1.14 ± 0.06 (14)	–1.18 ± 0.15 (0.06)
I128^3.46^A	3.81 ± 0.12 (154)	5.00 ± 0.13 (10,000)	0.06 ± 0.13 (1.1)^f^	4.90 ± 0.18 (13)	1.63 ± 0.17 (43)	–3
F353^6.44^A	5.57 ± 0.10 (2.7)	4.89 ± 0.07 (12,880)	0.10 ± 0.12 (1.3)^f^	5.13 ± 0.16 (7.4)	1.06 ± 0.21 (11)	–1.06 ± 0.21 (0.09)
N393^7.49^A	4.09 ± 0.09 (81)	5.85 ± 0.04 (1412)	–0.08 ± 0.10(0.8)^f^			

a The negative logarithm of the equilibrium dissociation constant of DA in the radioligand binding assay.

b The potency of DA in the functional assay.

c The negative logarithm of the equilibrium dissociation constant of the D2 PAM2 determined in the functional assays.

d Logarithm of the net cooperativity factor between the D2 PAM2 and DA determined in the functional assay.

e Estimate of the logarithm of the modulatory factor and orthosteric ligand efficacy determined in the functional assay.

f Value significantly different from that obtained in the control condition, one-way ANOVA, Dunnet’s post hoc test, *P* < 0.05.

g The affinity of DA and the fold shift in DA IC_50_ caused by 10 μM PAM at the WT or mutant SNAP-D_2S_R expressed in Flp-In-CHO cells were measured using a cell membrane [^3^H]­spiperone assay. The affinity of the D2 PAM2, its intrinsic efficacy, and cooperativity with DA at WT or mutant SNAP-D_2S_R expressed in Flp-In-CHO cells were measured in a functional cAMP assay using a BRET biosensor.

## Discussion

In this paper, we provide evidence that a small-molecule D_2_R PAM binds to a hitherto unexploited extrahelical pocket. Based on the results of our site-directed mutagenesis and MD experiments, this binding site lies in a cleft toward the intracellular aspect of the D_2_R defined by residues in TM1, TM7, and helix 8. While mutations can exert allosteric effects such that a mutation to a residue outside the binding pocket may alter the affinity of a ligand for the receptor, it is noteworthy that the mutation of residues within the identified allosteric pocket abolished the action of the PAM but had no effect on the affinity or potency of DA. Our simulations suggest the most favorable orientation of the modulator at the allosteric pocket and describe key interactions. They reveal the importance of the hydroxyl group interacting with the main chain kink at the conserved P7.50 residue, which displaces a water molecule that occupies this spot in an experimentally determined D_2_R structure. They also show that the bulky methoxy group fills the cavity between TM1 and TM7 and that the indole moiety is involved in π–π stacking interactions with H8 phenylalanine residues. The simulations reveal that the allosteric pocket is well-defined and relatively buried, especially when the palmitoylated C-terminal cysteine is considered.

A previous study used a probe-defined mapping protocol to predict an extracellular binding site for D2 PAM2 defined by residues at the extracellular side of TM2 and TM7 along with residues from ECL1 and 2.^21^ Our mutagenesis data found no significant effect on the affinity or modulatory action of the PAM when these residues were mutated to alanine. In a previous study, we provided evidence that the 1,2,3,4-tetrahydroquinolin-2-one secondary pharmacophore of the partial agonist aripiprazole extends into a secondary pocket defined by these TM2 and TM7 residues,^36^ in agreement with the pose of aripiprazole bound to serotonin 5HT_1a_R.^37^ In this paper, we also demonstrate that the PAM potentiates the efficacy of aripiprazole, an action difficult to reconcile with aripiprazole and D2 PAM2 binding to overlapping pockets defined by the same TM2 and TM7 residues.

Intracellular binding sites for small-molecular allosteric ligands have now been revealed for several class A GPCRs.^38,39^ This is not surprising given the allosteric communication between G protein binding at the intracellular surface of a GPCR and potentiating agonist binding at an extracellular orthosteric binding site. Indeed, monovalent nanobodies binding to intracellular sites of GPCRs have proven important tools with which to capture active agonist-bound receptor states in structural biology studies. Crystal structures of the CCR2 and CCR9 chemokine receptors and the β2 adrenergic receptor in complex with negative allosteric modulators revealed an overlapping intracellular pocket defined by residues from the intracellular ends of TM1, TM2, TM6, TM7, H8, and ICL1 and exposed to the cytosol.^40,41^ In comparison, the D2 PAM2 pocket is shifted away from the center of the helical bundle to include the receptor–lipid interface. A cryo-EM structure revealed that the D_1_R PAM, LY3154208, was bound in a lipid-embedded binding site created by predominantly hydrophobic residues from ICL2, TM3, and TM4.^25,42,43^ Comparison of this structure with D_1_R structures with only orthosteric agonists bound revealed rotamer changes in the side chains of W123^3.53^ and R130^ICL2^, such that the binding of LY3154208, by stabilizing the active conformation of TM3 and ICL2, acts to increase DA binding affinity and G protein coupling.^42,43^ Although the location of the pocket for the D2 PAM2 is different from that of LY3154208, there are similarities in the lipid-exposed nature of the pocket and their mechanism of action. Finally, a recent study has shown that a PAM (LUF6000) at the adenosine A_3_ receptor binds to an analogous extrahelical pocket defined by residues from H8, TM1, and TM7.^44^

In our experiments measuring D_2_R activation in a cAMP assay, we observe agonism from D2 PAM2 alone in conditions of high D_2_R expression levels. This agonism was relatively weak, being approximately 2-fold lower than that of the partial agonist aripiprazole. Nonetheless this suggests that D2 PAM2 binding stabilizes a conformation of the receptor that both increases efficacious agonist affinity to the orthosteric site and intracellular G protein coupling in a manner similar to the action of the LY3154208 at the D_1_R. This mechanism likely explains the action of the D2 PAM2 to increase the efficacy of D_2_R partial agonists by facilitating efficient G protein coupling. In agreement with this hypothesis, D2 PAM2 was able to rescue the efficacy of DA at mutant receptors in which key residues involved in the transition of an agonist-activated class A GPCR to an active state had been mutated to alanine.

This is further supported by the results of our MD simulations, where D2 PAM2 acts to maintain a receptor conformation that is favorable for G protein binding, even in the absence of an agonist. According to the simulations, TM7 and TM5 play the main role in this mechanism, which is consistent with a previous study on GPCR signaling.^45^ The intracellular part of TM7 contains the conserved NPxxY motif, where the tyrosine 7.53 constitutes a part of the tyrosine toggle switch and the asparagine 7.49 participates in the conserved Na^+^ binding pocket and the intrareceptor hydrogen bond network. The conformation of these residues governs receptor activation. Similarly, serine residues 5.42 and 5.43 in TM5 of the D_2_R have an important role in orthosteric ligand binding, as demonstrated by mutagenesis studies and the recently determined cryo-EM structure.^26,46,47^ Conformational changes of TM5 upon activation were also identified in experimental structures, in particular in the complex of D_2_R and Gi protein reconstituted into a membrane.^23^ In our simulations, TM5 motions were found to transmit an allosteric signal between the tyrosine toggle switch and the orthosteric binding pocket. Conformations of orthosteric binding pocket serines and tyrosine 5.58 of the toggle switch were mutually dependent, enabling two-way communication between the orthosteric binding site in the extracellular part of the receptor and the intracellular allosteric pocket/G protein binding interface.

Taken together, the simulations suggest that the D2 PAM2 stabilizes a receptor conformation that is similar to a G protein-bound active-state structure, providing a plausible explanation for the pharmacological data. Stabilization of the active state by D2 PAM2 will increase high-affinity DA binding and explain the increased slow dissociation phase of the labeled agonist PTHP-d1. Furthermore, stabilization of the fully active state of the receptor by the modulator would facilitate G protein recruitment and lead to increased efficacy of the partial agonists. Partial agonist binding does not result in a full receptor response, as these compounds stabilize receptor conformations that are suboptimal for recruitment of the intracellular signaling partners, which would be altered by D2 PAM2 cobinding. However, D2 PAM2 binding would not result in increased partial agonist affinity as the receptor conformation most suitable for binding of a partial agonist is not identical to the fully active conformation of the receptor. Thus, at mutations of transmission residues that reduced the efficacy of DA, the PAM acts to increase DA efficacy but not affinity.

This study adds to growing evidence that targeting intracellular pockets and/or lipid-exposed pockets is a viable but hitherto relatively underexploited approach to target GPCRs. D2 PAM2 has been shown to act to potentiate a subeffective dose of L-DOPA in a rodent model of Parkinson’s disease.^11^ D2 PAM2 displays subtype selectivity across the D_2_-like DRs, and as such, positive allosteric modulation represents a new approach toward the treatment of Parkinson’s disease either alone or in combination with subeffective doses of orthosteric agonists. This study, by providing insight into its binding mode and mechanism of action, may facilitate such efforts.

## Methods

### Materials

The biosensor vector pcDNA3L-His-CAMYEL was purchased from ATCC. Dulbecco’s modified Eagle’s medium (DMEM), hygromycin B, and Flp-In-CHO cells were purchased from Invitrogen (Carlsbad, CA). Fetal bovine serum (FBS) was purchased from Thermo Trace (Melbourne, VIC, Australia). [^3^H]­Spiperone, [^3^H]­raclopride, AlphaScreen reagents, Ultima gold scintillation cocktail, 384-well OptiPlates, and 384-well ProxiPlates were purchased from Revvity (Boston, MA). The D2 PAM2 and [^3^H]­rotigotine were a gift from UCB Pharma. All the other reagents were purchased from Sigma-Aldrich (Castle Hill, NSW, Australia).

### Molecular Biology

cDNA in pcDNA3.1+ encoding the short isoform of the wild-type human dopamine D_2_ receptor containing an N-terminal SNAP-tag was obtained from Cisbio (Bagnols-sur-Cèze, France). cDNAs in pcDNA3.1+ encoding the long isoform of the wild-type human dopamine D_2_ receptor, the dopamine D_3_ receptor, and the dopamine D_4.4_ receptor were obtained from the cDNA Resource Center (www.cdna.org). Enzymes for DNA cloning were sourced from New England Biolabs (Ipswich, MA). Oligonucleotides were supplied by Integrated DNA Technologies (Singapore). The receptor construct in pcDNA3.1+ was transferred into a modified pcDNA5 (the CMV promoter was replaced by an EF-1α promoter) by restriction enzyme cloning. Desired mutations were introduced by PCR site-directed mutagenesis. The DNA constructs of chimeric receptors were created according to a method described by Ko and Ma, facilitated by the type II restriction enzyme *Bsa*I.^48^

### Cell Lines and Transfection

Flp-In-CHO cells stably expressing the CAMYEL biosensor were maintained in a DMEM/F-12 medium supplemented with 5% fetal bovine serum and 0.5 mg/mL G418 at 37 °C in a humidified incubator containing 5% CO_2_. The cells were transfected with the pOG44 vector encoding Flp recombinase and the modified pcDNA5 vector encoding the wild-type or mutant DRs at a mass ratio of 9:1 using linear 25 kDa polyethylenimine (Polysciences, PA) as a transfection reagent. Twenty-four hours after transfection, the cells were subcultured and selected with 700 μg/mL HygroGold (Invivogen) to obtain cells stably expressing the receptors.

### ELISA

Cells were plated into 48-well culture plates at a density of 125,000 cells/well. The following day, the cells were washed with Tris-buffered saline (TBS, pH 7.4) and fixed in 3.7% v/v formaldehyde for 30 min. Following another wash with TBS, the cells were blocked on a shaker at 4 °C using blocking buffer (TBS, 1% fat-free milk). On day three, the blocking buffer was discarded, and the cells were incubated with the primary antibody (1:1000, SNAP antibody, Thermo Fisher) for 4 h at RT. After washing with TBS, the cells were incubated for 2 h (RT) with the secondary antibody (1:2000, HRP-linked antirabbit IgG, Cell Signaling Technology). The peroxidase substrate (SIGMAFAST OPD) was prepared according to the instruction of the supplier and added after washing the cells with TBS. The reaction was terminated by the addition of 1M HCl. The colored reaction product was detected at 490 nm in an EnVision multilabel plate reader (Revvity). The absorbance values for cells stably expressing the mutant were normalized to those obtained for untransfected CHO cells (0%) and cells expressing WT SNAP-D_2S_R (100%).

For ELISA experiments measuring D2R internalization, CHO cells stably overexpressing GRK2 and transiently expressing HA-D2L-Rluc were plated into 48-well culture plates at a density of 125,000 cells/well. The following day, cells were stimulated with DA and D2 PAM2 for 1 h and then cells were washed with Tris-buffered saline (TBS, pH 7.4) and fixed in 3.7% v/v formaldehyde for 30 min. Following another wash with TBS, the cells were blocked on a shaker o/n at 4 °C using blocking buffer (TBS, 1% fat-free milk). On day three, surface human influenza hemagglutinin (HA)-tagged receptors were detected using the HA-7 mouse anti-HA antibody (1:1000, Sigma-Aldrich, Sydney, Australia), followed by HRP-conjugated goat antimouse IgG (1:2000, Sigma-Aldrich). After washing with TBS, the peroxidase substrate SIGMAFAST OPD was added and the reaction was terminated by the addition of 1 M HCl. The colored reaction product was detected at 490 nm in a multilabel plate reader (EnVision, Revvity).

#### ERK1/2 Phosphorylation Assay

Flp-In-CHO cells stably expressing the D_2L_R were seeded into 96-well plates at a density of 50,000 cells/well. After 5 h, cells were washed with phosphate-buffered saline (PBS) and incubated in serum-free DMEM overnight before assaying. Initially, time-course experiments were conducted at least twice for each ligand to determine the time required to maximally promote ERK1/2 phosphorylation via the dopamine D_2L_R. Interaction studies were performed using varying concentrations of the test ligand and increasing concentrations of dopamine at 37 °C with a stimulation time of 5 min. Stimulation of the cells was terminated by removing the medium followed by the addition of 100 μL of SureFire lysis buffer (PerkinElmer) to each well. The plate was shaken for 5 min at RT before 5 μL of the lysates was transferred to a white 384-well ProxiPlate (PerkinElmer). Then, 8 μL of a 240:1440:7:7 mixture of Surefire activation buffer:Surefire reaction buffer:AlphaScreen acceptor beads:AlphaScreen donor beads was added to the samples and incubated in the dark at 37 °C for 1.5 h. Plates were read using a Fusion plate reader.

### Bioluminescence Resonance Energy Transfer (BRET) Assays

#### cAMP

Flp-In-CHO cells stably coexpressing the CAMYEL biosensor and DRs were plated into white 96-well CulturPlates (Revvity) and grown overnight. The cells were equilibrated in Hank’s balanced salt solution (HBSS) at 37 °C (or 25 °C when indicated) before starting the experiment. The cells were costimulated with the agonists and 10 μM forskolin for the indicated time frames when the BRET readings were captured. Coelenterazine (Nanolight Technology, AZ) was added at a final concentration of 5 μM at least 3 min prior to measurement. The signals were detected at 445–505 and 505–565 nm by using a LUMIstar Omega instrument (BMG LabTech, Offenburg, Germany). Net BRET was determined by subtraction of the vehicle control coadded with 10 μM forskolin. For phenoxybenzamine alkylation experiments, the cells were treated with 0.1, 0.3, or 1 μM phenoxybenzamine (Sigma-Aldrich) diluted in HBSS and equilibrated at 37 °C for 30 min prior to the commencement of the assay.

#### β-Arrestin-2 Recruitment

Flp-In-CHO cells were seeded at a density of 2,000,000 cells per 10 cm dish and were transfected the following day using polyethylenimine as a transfection reagent. To assess β-arrestin-2 recruitment to the D_2L_R, the cells were transfected with 1 μg of Rluc8-tagged D_2L_R, 4 μg of YFP-β-arrestin-2, and 2 μg of GRK2. Twenty-four hours after transfection, the cells were plated into 96-well CulturPlates (PerkinElmer) and grown overnight. The cells were equilibrated in Hank’s balanced salt solution at 37 °C before starting the experiment. The cells were incubated with coelenterazine (Promega) at a final concentration of 5 μM for 5 min followed by stimulation with the agonists for an additional 5 min before the BRET readings were captured. The signals were detected at 445–505 and 505–565 nm using a LUMIstar Omega instrument (BMG LabTech, Offenburg, Germany). Net BRET was determined by subtraction of the vehicle control from the agonist-induced response.

### Membrane Preparation

Flp-In-CHO cells stably expressing the wild-type or mutant DRs were grown to 90% confluence in 175 cm^2^ cell culture flasks. The cells were harvested in PBS containing 2 mM EDTA and centrifuged at 300*g* for 3 min. The resulting pellet was resuspended in ice-cold assay buffer (20 mM HEPES, 100 mM NaCl, 6 mM MgCl_2_, 1 mM EGTA, and 1 mM EDTA, pH 7.4), and the centrifugation step was repeated. The intact cell pellet was then resuspended in assay buffer and homogenized using a Polytron homogenizer. After centrifugation (1000*g*, 10 min), the pellet was discarded, and the supernatant was recentrifuged at 30,000*g* for 1 h at 4 °C using a Sorvall Evolution RC ultracentrifuge (Thermo Scientific). The resulting pellet was resuspended in assay buffer and stored in 250 μL aliquots at −80 °C. Membrane protein concentration was determined using a Pierce’s BCA protein assay kit (Thermo Fisher, Melbourne).

### Radioligand Binding Assays

[^3^H]­Spiperone binding experiments using cell membranes were conducted in a 200 μL reaction volume in assay buffer (20 mM HEPES, 100 mM NaCl, 6 mM MgCl_2_, 1 mM EGTA, and 1 mM EDTA, pH 7.4) containing 100 μM GppNHp and 0.1% ascorbic acid in 96-well plates. The buffer for the [^3^H]­rotigotine binding assay contained 50 mM Tris-HCl pH 7.4 and 2 mM MgCl_2_. In all cases, nonspecific binding was determined in the presence of 10 μM haloperidol. To obtain affinity estimates of unlabeled agonists, competition binding experiments were performed at equilibrium. The ability of increasing concentrations of the agonists to compete with 1 nM [^3^H]­raclopride for binding to wild-type or mutant SNAP-D_2S_R was tested. The binding experiments with [^3^H]­raclopride were conducted at 25 °C, whereas those with [^3^H]­spiperone were conducted at 37 °C. The membranes (5 μg) were incubated with the drugs for 3 h at 37 °C ([^3^H]­spiperone) or 25 °C ([^3^H]­raclopride). After this incubation period, bound and free radioligands were separated by fast-flow filtration through UniFilter-96 GF/B plates using a MicroBeta FilterMate harvester (Revvity) followed by three washes with ice-cold 0.9% NaCl. Filter bound radioactivity was detected as a chemiluminescence after the addition of MicroScint-20 (Revvity) in a MicroBeta plate counter (Revvity).

For [^3^H]­raclopride binding analysis using intact cells, 50,000 cells/well were seeded into an isoplate (Revvity) overnight. The binding reaction was conducted in HBSS for 1 h at 37 °C. The adherent cells were washed two times with cold 0.9% NaCl solution, and 100 μL/well scintilation liquid was added to the plate. The remaining radioactivity was detected in a MicroBeta counter.

#### [^35^S]­GTPγS Binding Assay

Cell membranes of Flp-In-CHO/D_2L_R (5 μg of the protein) were equilibrated for 60 min at 30 °C with varying concentrations of ligands in a buffer containing 20 mM HEPES, 10 mM MgCl_2_, 150 mM NaCl, and 5 μg/mL saponin, pH 7.4, containing 3 μM. [^35^S]­GTPγS (0.1 nM) was added to a final volume of 0.2 mL, and membranes were incubated for a further 60 min at 30 °C. The [^35^S]­GTPγS binding reaction was terminated by rapid filtration with a MicroBeta FliterMate harvester (Revvity) onto 96-well GF/C filter plates followed by three washes with ice-cold 0.9% NaCl. Bound radioactivity was measured on a MicroBeta microplate counter (PerkinElmer). Data were normalized to the maximal response of dopamine under the control condition. For the pertussis-treated condition, Flp-In-CHO/D_2L_R cells were treated with a 125 ng/mL pertussis toxin overnight before membrane preparation. Urea pretreatment of Flp-Ii CHO/D_2L_R membranes was performed according to a method described by May et al.^49^

### SNAP-tag Labeling and Membrane Preparation

SNAP-tag labeling and membrane preparations were performed as previously described with minor modifications. Flp-In-CHO cells stably expressing the SNAP-D_2S_R were cultured in DMEM/F-12, 10% FBS, and 700 μg/mL hygromycin B. Cells were grown to reach 80–90% confluency in T175 flasks before labeling. Cell culture media was removed, and receptors were labeled by incubation in 10 mL of Tag-lite buffer (Cisbio) containing 2 nmol of SNAP-Lumi4-Tb (Cisbio) in a 5% CO_2_ incubator for 1 h at 37 °C. SNAP-Lumi4-Tb was then removed, and cells were washed and harvested in DPBS. Cells were collected by centrifugation at 300*g* for 5 min, and cell pellets were stored at −80 °C before membrane preparations.

Membrane preparations were performed on ice with ice-cold buffers to maintain the D2SR integrity. Cell pellets were resuspended in 10 mL of buffer 1 (10 mM HEPES and 10 mM EDTA, pH 7.4). Cells were subsequently homogenized with an IKA Works T10 Ultra-Turrax homogenizer on setting 4–10 short pulses. Homogenates were then made up to 20 mL with buffer 1 followed by centrifugation at 48,000*g* in an Avanti J-25 ultracentrifuge with a JA25.50 rotor (Beckman) for 30 min at 4 °C. After centrifugation, the supernatant was discarded. The pellet was resuspended in 20 mL of buffer 1 and recentrifuged as before. The supernatant was then discarded, and the final membrane pellet was resuspended in 10 mM HEPES and 0.1 mM EDTA, and pH 7.4. Membranes were aliquoted and stored at −80 °C. Membrane protein concentration was determined using the bicinchoninic acid assay (Sigma-Aldrich).

### Time-Resolved Fluorescence Resonance Energy Transfer (TR-FRET) Kinetic Binding Assays

TR-FRET binding assays were performed in white opaque 384-well OptiPlates (PerkinElmer) at 37° C. SNAP-D_2S_R cell membranes and all ligands were diluted in Hanks’ balanced salt solution (HBSS) (Sigma-Aldrich) supplemented with 20 mM HEPES and 0.02% w/v pluronic acid, pH 7.4. Each well contained 2 μg of cell membrane protein in 2.5 μg/mL saponin, 50 nM PPHT-d1 (Cisbio), varying concentrations of the D2 PAM2, and either the presence or absence of 100 μM Gpp­(NH)­p. Nonspecific binding for each condition was determined in the presence of 10 μM haloperidol. The microplate was incubated for 1 h at 37 °C in a total volume of 40 μL. PPHT-d1 dissociation was then initiated by addition of 10 μL of spiperone to make a final concentration of 24 μM.

Time-resolved fluorescence was detected using a PHERAstar FS microplate reader (BMG Labtech) at 37 °C. Terbium cryptate was excited at 337 nm, and emission was detected at 620 nm (donor) and 665 nm (acceptor) using the HTRF filter module. The cycle time was set to 10 s with 6 laser flashes per well per cycle (integration start: 60 μs, integration time: 400 μs). HTRF ratios were determined by dividing the 665 nm counts (acceptor) by the 620 nm counts (donor) multiplied by 10,000. The HTRF ratios were then subtracted by the nonspecific binding HTRF ratios to give specific binding. The specific binding was then baseline-normalized over time whereby wells with PPHT-d1 absent were defined as 0% and wells containing 50 nM PPHT-d1 (absence of competing spiperone) were defined as 100%.

### Data Analysis

#### Radioligand Binding Data

For radioligand saturation binding data, the following equations were globally fitted to total binding (1) and nonspecific binding (2) data:

$$Y=\frac{B_{\max}[A]}{[A]+K_{A}}+\mathrm{NS}[A]$$ 1

Y=NS[A] 2

where *Y* is radioligand binding, *B*_max_ is the total receptor density, [A] is the free radioligand concentration, *K*_A_ is the equilibrium dissociation constant of the radioligand, and NS is the fraction of nonspecific radioligand binding.

Competition binding curves between [^3^H]­spiperone or [^3^H]­raclopride and dopamine in the absence or presence of D2 PAM2 were fitted to a one-site binding equation:

$$Y=\frac{B_{\max}[A]}{[A]+\left(\frac{K_{A}K_{B}}{\alpha [B]+K_{B}}\right)\left(1+\frac{\left[I\right]}{K_{I}}+\frac{\left[B\right]}{K_{B}}+\frac{\alpha^{\prime }[I][B]}{K_{I}K_{B}}\right)}$$ 3

where *Y* is the percentage (vehicle control) binding, [A], [B], and [I] are the concentrations of [^3^H]­spiperone, D2 PAM2, and DA, respectively, *K*_A_ and *K*_B_ are the equilibrium dissociation constants of [^3^H]­spiperone and D2 PAM2, respectively, *K*_I_ is the equilibrium dissociation constant of dopamine and α′ and α represent the cooperativity between the D2 PAM2 and [^3^H]­spiperone/[^3^H]­raclopride or dopamine, respectively. Values of α (or α′) >1 denote positive cooperativity; values <1 (but >0) denote negative cooperativity, and values = 1 denote neutral cooperativity.

The concentration of the ligand that inhibited half of the [^3^H]­raclopride binding (IC_50_) was determined using the following equation:

$$Y=\frac{\mathrm{bottom}+(\mathrm{top}-\mathrm{bottom})}{1+10^{(X-\log\;{\mathrm{IC}}_{50})n_{H}}}$$ 4

where *Y* denotes the percentage-specific binding, top and bottom denote the maximal and minimal asymptotes, respectively, IC_50_ denotes the X-value when the response is midway between bottom and top, and *n*_H_ denotes the Hill slope factor. IC_50_ values obtained from the inhibition curves were converted to *K_i_* values using the Cheng and Prusoff equation.

#### Functional Data

Dose–response curves were fitted using the following three-parameter equation:

$$\mathrm{response}=\mathrm{bottom}+\frac{\mathrm{top}-\mathrm{bottom}}{1+10^{\left(\log{\mathrm{EC}}_{50}-\log[A]\right)}}$$ 5

where top and bottom represent the maximal and minimal asymptote of the dose response curve, [A] is the molar concentration of the agonist, and EC_50_ is the molar concentration of the agonist required to give a response half-way between bottom and top.

Functional data describing the interaction between the D2 PAM2 and orthosteric agonists were analyzed using a complete operational model of allosterism and agonism according to the equation:

$$E=\left\{E_{m}(\tau_{A}[A](K_{B}+\alpha \beta [B])+\tau_{B}[B]K_{A}{)}^{n_{H}}\right\}/\left\{([A]K_{B}+K_{A}K_{B}+K_{A}[B]+\alpha [A][B]{)}^{n_{H}}+(\tau_{A}[A](K_{B}+\alpha \beta [B]+\tau_{B}[B]K_{A}{)}^{n_{H}}\right\}$$ 6

where *E*_m_ is the maximum possible cellular response, [A] and [B] are the concentrations of orthosteric and allosteric ligands, respectively, *K*_A_ and *K*_B_ are the equilibrium dissociation constant of the orthosteric and allosteric ligands, respectively, τ_A_ and τ_B_ are operational measures of orthosteric and allosteric ligand efficacy (which incorporate both signal efficiency and receptor density), respectively, α is the binding cooperativity parameter between the orthosteric and allosteric ligand, and β denotes the magnitude of the allosteric effect of the modulator on the efficacy of the orthosteric agonist.

### Molecular Modeling and Molecular Dynamics Simulations

#### Protein Preparation

From the D_2_R structures available in the PDB database, the 6VMS structure was selected.^23^ The structure was resolved in a lipid bilayer and includes a complete C-terminus. The construct includes also a shortened ICL3 that was shown to be functional in vitro. Protein protonation states were derived from PROPKA.^50^ In particular, the Asp 2.50 side chain was protonated. A C-terminal cysteine residue was palmitoylated, as indicated in the literature.^28^ Two versions of the simulation box were prepared: with and without truncated α subunits of G protein bound at the intracellular part. Both versions used the same membrane structure. The smaller box was prepared by decreasing the amount of the aqueous solvent.

#### Docking

An initial putative D_2_-PAM complex structure was prepared with Glide and Induced Fit Docking modules of the Schrödinger suite.^51^ The initial structure of the D_2_-rotigotine complex was prepared using the 8IRS PDB structure.^24^

#### Simulation Box Setup

An asymmetric, native-like membrane composed of nine types of lipids, including cholesterol, sphingomyelin, palmitoyl, oleyl, linoleoyl lipid chains, and choline, ethanolamine, glycerol, and serine head groups, was built with CHARMM-GUI.^52^ A simulation box was filled with water and 0.15 M NaCl. A TIP3P water model was used. A set of compatible force fields used in the simulations included Amber03, GAFF2, and Slipids for protein, ligand, and membrane, respectively.^53,54^ Ligand charges were derived using PyRED.^55^ Gromacs topologies of ligands were generated using ACPYPE.^56^ Thioester bond parameters in palmitoylated cysteine were based on previously published works.^57–59^ Membranes were equilibrated with short, 1 ns NPT simulations using Berendsen pressure coupling for quick adjustment of the simulation box dimensions, followed by 300 ns of NPT simulation using a Parrinello–Rahman barostat. Position restraints of 1000 kJ/mol nm^2^ were applied on the protein backbone during the equilibration. The resultant structures were used as starting points for all simulations using six different random seeds for temperature generation. A Nose–Hoover thermostat and Parrinello–Rahman semi-isotropic pressure coupling were used in production runs.

#### Metadynamics

During the enhanced sampling simulations, the PAM was restrained within the area of binding by walls of potential equal to 3500 kJ/mol/nm^2^ using the UPPER_WALLS action of Plumed. The area was defined as the distance below 5 Å between the center of mass of the PAM and the center of mass of the main chain atoms of F1.48, I7.51, and F8.54 residues. Collective variables were defined as the distance between the PAM and proline 7.50 and torsion between the modulator and TM7. Gaussians were deposited every 1000 steps on a grid, with bin sizes of 0.1 nm and 0.43 radian, respectively. Adaptive bias was applied, with a Δ*T* value of 1236 K and an initial Gaussian height of 0.5 kJ/mol. A multiple-walker approach was used. Each of five walkers was simulated for ca. 380 ns. Convergence of the enhanced sampling simulations was assessed by comparison of the free energy profiles at different simulation times (Supporting Information S15). Gromacs 2023 ^60^ patched with Plumed 2.9.0 ^62^ was used as an MD engine. Ligand poses corresponding to the free energy minimum were extracted and clustered with a single linkage algorithm. For additional validation, the central frames of the largest clusters were investigated during short MD simulations. Only the frame corresponding to the largest cluster was found to be stable. The pose extracted from that frame was used further in all-atom MD.

#### All-Atom Unbiased Molecular Dynamics Simulations

All-atom unbiased MD simulations were performed with Gromacs 2023. Every system was simulated in six replicas. Each replica was started with a different random seed for velocity generation. The same set of six seeds was used in all systems to ensure that differences between complexes are due to different ligand configurations. Each replica was simulated for 1 μs.

PCA was performed with Gromacs tools gmx covar and gmx anaeig. PCA was run in a common subspace on concatenated trajectories. Several different selections were used to remove the noise.

Trajectory analyses were performed with the open-source, community-developed PLUMED library,^61^ version 2.9,^62^ and with ProLIF tool, version 2.0.3.^63^ Plots and heat maps were generated with Gnuplot,^64^ and interaction barcodes were visualized with Matplotlib.^65^ Molecular visualizations were made using PyMOL.^66^

## Supplementary Material



## Notes

(S1) Structures of the dopamine D_2_ receptor agonists (dopamine and rotigotine) and positive allosteric modulators (D2 PAM1 and D2 PAM2); (S2) D2 PAM displaying probe dependence; (S3) D2 PAM2 modulating [^3^H]­raclopride binding; (S4) removal of heterotrimeric G proteins ablating the response of dopamine; (S5) D2 PAM2 increasing the affinity of [^3^H]­rotigotine; (S6) D2 PAM increasing the percentage of slow-phase agonist dissociation; (S7) action of D2 PAM2 to modulate the action of DA at the D2R in different signaling assays; (S8) parameters describing the activity of D2 PAM2 across different end points; (S9) D2 PAM displaying subtype selectivity; (S10) mutation of residues within an intracellular pocket of the D2R abrogating the action of the D2 PAM; (S11) cell surface expression of D2R mutants; (S12) D2 PAM unaffected by mutation of extracellular binding pocket residues (figures); (S13) D2 PAM unaffected by mutation of extracellular binding pocket residues (parameters); (S14) differences in the allosteric pocket between D2-4Rs; (S15) convergence of the metadynamics simulation; (S16) effect of D2 PAM2 on DA binding inhibited by mutation of receptor activation residues; (S17) protein-modulator interaction barcodes in rotigotine- and PAM2-bound receptor simulations; (S18) protein-modulator interaction barcodes in PAM2-bound receptor simulations; (S19) RMSD of the heavy atoms of the receptor in all simulations; (S20) RMSF of the receptor protein in all simulations (PDF)

## †

Division of Medicinal Chemistry, Amsterdam Institute of Molecular and Life Sciences (AIMMS), Vrije Universiteit Amsterdam, De Boelelaan 1108, 1081 HZ Amsterdam, The Netherlands

## ‡

OMass Therapeutics, ARC, Oxford OX4 2GX, United Kingdom

## §

Kesmalea Therapeutics, 8 Bloomsbury Street, London WC1B 3SR, United Kingdom

## ∥

Eli Lilly and Company, Lilly Biotechnology Center, 10290 Campus Point Dr., San Diego, California 92121, United States

## References

[1] Beaulieu J.-M. M., Gainetdinov R. R. (2011). The physiology, signaling, and pharmacology
of dopamine receptors. Pharmacol. Rev..

[2] Blandini F., Armentero M.-T. (2014). Dopamine
receptor agonists for Parkinson’s disease. Expert Opinion on Investigational Drugs.

[3] Seeman P. (2006). Targeting
the dopamine D2 receptor in schizophrenia. Expert
Opinion on Therapeutic Targets.

[4] Agid Y. (1991). Parkinson’s
disease: pathophysiology. Lancet.

[5] Agid Y. (2007). How can drug discovery for psychiatric disorders be
improved?. Nat. Rev. Drug Discovery.

[6] Obeso J. A., Olanow C. W., Nutt J. G. (2000). Levodopa motor complications in Parkinson’s
disease. Trends in Neurosciences.

[7] Voon V., Mehta A. R., Hallett M. (2011). Impulse control
disorders in Parkinson’s
disease: recent advances. Current Opinion in
Neurology.

[8] Christopoulos A. (2014). International Union
of Basic and Clinical Pharmacology. XC. multisite
pharmacology: recommendations for the nomenclature of receptor allosterism
and allosteric ligands. Pharmacol. Rev..

[9] Wootten D., Christopoulos A., Sexton P. M. (2013). Emerging paradigms in GPCR allostery:
implications for drug discovery. Nat. Rev. Drug
Discovery.

[10] Jeffrey
Conn P., Christopoulos A., Lindsley C. W. (2009). Allosteric modulators
of GPCRs: a novel approach for the treatment of CNS disorders. Nat. Rev. Drug Discovery.

[11] Wood M. (2016). In Vitro and In Vivo Identification of Novel Positive Allosteric
Modulators of the Human Dopamine D2 and D3 Receptor. Molecular Pharmacology.

[12] Canals M. (2012). A Monod-Wyman-Changeux Mechanism Can Explain G Protein-coupled Receptor
(GPCR) Allosteric Modulation. J. Biol. Chem..

[13] DeVree B. T. (2016). Allosteric coupling
from G protein to the agonist-binding pocket
in GPCRs. Nature.

[14] Kara E., Lin H., Strange P. G. (2010). Co-operativity
in agonist binding at the D2 dopamine
receptor: evidence from agonist dissociation kinetics. Journal of neurochemistry.

[15] Herenbrink C. K. (2016). The role of kinetic
context in apparent biased agonism at GPCRs. Nat. Commun..

[16] Sykes D. A. (2017). Extrapyramidal side effects of antipsychotics
are linked to their
association kinetics at dopamine D2 receptors. Nat. Commun..

[17] Löber S., Hübner H., Tschammer N., Gmeiner P. (2011). Recent advances in
the search for D3- and D4-selective drugs: probes, models and candidates. Trends in Pharmacological Sciences.

[18] Wang S. (2018). Structure of the D2
dopamine receptor bound to the atypical antipsychotic
drug risperidone. Nature.

[19] Wang S. (2017). D4 dopamine receptor high-resolution structures enable the discovery
of selective agonists. Science.

[20] Chien E. Y. T. (2010). Structure of the Human
Dopamine D3 Receptor in Complex
with a D2/D3 Selective Antagonist. Science.

[21] Ciancetta A. (2021). Probe Confined Dynamic
Mapping for G Protein-Coupled Receptor Allosteric
Site Prediction. ACS Cent. Sci..

[22] Fan L. (2020). Haloperidol bound D2
dopamine receptor structure inspired the discovery
of subtype selective ligands. Nat. Commun..

[23] Yin J., Chen K. Y. M., Clark M. J., Hijazi M., Kumari P., Bai X. C., Sunahara R. K., Barth P., Rosenbaum D. M. (2020). Structure
of a D2 dopamine receptor-G-protein complex in a lipid membrane. Nature.

[24] Xu P. (2023). Structural genomics
of the human dopamine receptor system. Cell
Res..

[25] Zhuang Y. (2021). Structural insights into the human D1 and D2 dopamine receptor signaling
complexes. Cell.

[26] Knight K. M. (2024). A neurodevelopmental disorder mutation locks
G proteins in the transitory
pre-activated state. Nat. Commun..

[27] Im D. (2020). Structure of the dopamine D2 receptor in complex with
the antipsychotic
drug spiperone. Nat. Commun..

[28] Ebersole B. (2015). Effect of C-Terminal S-Palmitoylation on D2 Dopamine Receptor Trafficking
and Stability. PLoS One.

[29] Trzaskowski B. (2012). Action of Molecular
Switches in GPCRs - Theoretical and Experimental
Studies. Curr. Med. Chem..

[30] Dror R. O. (2011). Activation mechanism
of the β2-adrenergic receptor. Proc. Natl.
Acad. Sci. U. S. A..

[31] Powers A. S. (2024). A non-canonical mechanism of GPCR activation. Nat. Commun..

[32] Latorraca N. R., Venkatakrishnan A. J., Dror R. O. (2017). GPCR Dynamics: Structures in Motion. Chem. Rev..

[33] Urizar E. (2005). An activation switch
in the rhodopsin family of G protein-coupled
receptors: the thyrotropin receptor. J. Biol.
Chem..

[34] Michino M., Free R. B., Doyle T. B., Sibley D. R., Shi L. (2015). Structural
basis for Na­(+)-sensitivity in dopamine D2 and D3 receptors. Chemical communications (Cambridge, England).

[35] Katritch V. (2014). Allosteric sodium in
class A GPCR signaling. Trends in Biochemical
Sciences.

[36] Herenbrink C. K. (2019). Molecular Determinants of the Intrinsic Efficacy of the Antipsychotic
Aripiprazole. ACS Chem. Biol..

[37] Xu P. (2021). Structural insights
into the lipid and ligand regulation of serotonin
receptors. Nature.

[38] Zhang L., Mobbs J. I., May L. T., Glukhova A., Thal D. M. (2023). The impact
of cryo-EM on determining allosteric modulator-bound structures of
G protein-coupled receptors. Curr. Opin. Struct.
Biol..

[39] Zacarías N. V.
O., Lenselink E. B., IJzerman A. P., Handel T. M., Heitman L. H. (2018). Intracellular
Receptor Modulation: Novel Approach to Target GPCRs. Trends Pharmacol. Sci..

[40] Zheng Y. (2016). Structure of CC Chemokine
Receptor 2 with Orthosteric and Allosteric
Antagonists. Nature.

[41] Oswald C. (2016). Intracellular allosteric
antagonism of the CCR9 receptor. Nature.

[42] Teng X. (2022). Ligand recognition and
biased agonism of the D1 dopamine receptor. Nat. Commun..

[43] Zhuang Y. (2021). Mechanism of dopamine
binding and allosteric modulation of the human
D1 dopamine receptor. Cell Res..

[44] Fisher C. L. (2024). Extrahelical binding
site for a 1H-imidazo­[4,5-c]­quinolin-4-amine
A3 adenosine receptor positive allosteric modulator on helix 8 and
distal portions of transmembrane domains 1 and 7. Mol. Pharmacol..

[45] Ramos-Gonzalez N., Paul B., Majumdar S. (2023). IUPHAR themed
review: Opioid efficacy,
bias, and selectivity. Pharmacol. Res..

[46] Mansour A. (1992). Site-directed mutagenesis
of the human dopamine D2 receptor. Eur. J. Pharmacol.:
Mol. Pharmacol..

[47] Wiens B. L., Nelson C. S., Neve K. A. (1998). Contribution of
Serine Residues to
Constitutive and Agonist-Induced Signaling via the D2SDopamine Receptor:
Evidence for Multiple, Agonist-Specific Active Conformations. Mol. Pharmacol..

[48] Ko J.-K., Ma J. (2005). A rapid and efficient
PCR-based mutagenesis method applicable to
cell physiology study. Am. J. Physiol.-Cell
Physiol..

[49] May L. T., Sexton P. M., Christopoulos A. (2005). Effects of urea pretreatment on the
binding properties of adenosine A1 receptors. British Journal of Pharmacology.

[50] Olsson M. H. M., So̷ndergaard C. R., Rostkowski M., Jensen J. H. (2011). PROPKA3: Consistent Treatment of
Internal and Surface
Residues in Empirical pK a Predictions. J. Chem.
Theory Comput..

[51] Schrödinger Release 2020–4: Schrödinger, LLC New York, NY, USA, 2020; Schrödinger, LLC: New York, NY, 2020.

[52] Lee J. (2016). CHARMM-GUI Input Generator for NAMD, GROMACS, AMBER,
OpenMM, and
CHARMM/OpenMM Simulations Using the CHARMM36 Additive Force Field. J. Chem. Theory Comput..

[53] Jämbeck J. P. M., Lyubartsev A. P. (2013). Another Piece of the Membrane Puzzle:
Extending Slipids Further. J. Chem. Theory Comput..

[54] He X., Man V. H., Yang W., Lee T.-S., Wang J. (2020). A fast and
high-quality charge model for the next generation general AMBER force
field. J. Chem. Phys..

[55] Wang, F. ; Becker, J. P. ; Dupradeau, P. C. and F, Y. & Y, F. R.E.D.Python: Object oriented programming for Amber force fields. In Université de Picardie–Jules Verne; Sanford Burnham Prebys Medical Discovery Institute: (2013).

[56] Sousa
da Silva A. W., Vranken W. F. (2012). ACPYPE - AnteChamber PYthon Parser
interfacE. BMC Res. Notes.

[57] Nagy P. I., Tejada F. R., Sarver J. G., Messer W. S. (2004). Conformational Analysis
and Derivation of Molecular Mechanics Parameters for Esters and Thioesters. J. Phys. Chem. A.

[58] Oda A., Fukuyoshi S., Nakagaki R., Takahashi O. (2013). Determination
of AMBER Force Field Parameters for Thioester by Quantum Chemical
Calculations. Chem. Lett..

[59] Zacharias D. E., Murray-Rust P., Preston R. M., Glusker J. P. (1983). The geometry of
the thioester group and its implications for the chemistry of acyl
coenzyme a. Arch. Biochem. Biophys..

[60] Hess B., Kutzner C., van der
Spoel D., Lindahl E. (2008). GROMACS 4: Algorithms
for Highly Efficient, Load-Balanced, and Scalable Molecular Simulation. J. Chem. Theory Comput..

[61] Bonomi M. (2019). Promoting transparency and reproducibility in enhanced molecular
simulations. Nat. Methods.

[62] Tribello G. A., Bonomi M., Branduardi D., Camilloni C., Bussi G. (2014). PLUMED 2: New feathers for an old
bird. Comput.
Phys. Commun..

[63] Bouysset C., Fiorucci S. (2021). ProLIF: a library to encode molecular
interactions
as fingerprints. J. Cheminf..

[64] Williams, T. ; Al, C. K. Gnuplot Version 5.4.

[65] Hunter J. D. (2007). Matplotlib:
A 2D Graphics Environment. Comput. Sci. Eng..

[66] Schrödinger LLC The PyMOL Molecular Graphics System, Version∼1.8.

