# Supplementary material for: Identification of a Lipid-Exposed Extrahelical Binding Site for Positive Allosteric Modulators of the Dopamine D2 Receptor
Source: ACS Chem Neurosci. 2025 May 15;16(12):2295–311. doi: 10.1021/acschemneuro.5c00105 (PMC12186844; doi:10.1021/acschemneuro.5c00105)
Supplement: Supplementary file 1 [file cn5c00105_si_001.pdf]

Identification of a lipid exposed extrahelical binding site for positive allosteric modulators of the dopamine D2 receptor

Authors: Herman D. Lim^[,1,2], Damian Bartuzi^[,3,4], Alastair C Keen[1,5,6], Caroline Rauffenbart[7,8], Jacqueline Glenn[5,6], Steven J. Charlton[5,6,9], Silvia Lovera[10,11], Zara A. Sands[10,12], Ali Ates[10], Martyn Wood[10], Meritxell Canals[5,6], Jonathan A. Javitch[7,8], Jens Carlsson*[,3], J. Robert Lane*[,5,6]

[1]Drug Discovery Biology, Monash Institute of Pharmaceutical Sciences, Monash University (Parkville campus), 399 Royal Parade, Parkville, VIC 3052, Australia

[2]Current address: Division of Medicinal Chemistry, Amsterdam Institute of Molecular and Life Sciences (AIMMS), Vrije Universiteit Amsterdam, De Boelelaan 1108, 1081 HZ Amsterdam, The Netherlands

[3]Science for Life Laboratory, Department of Cell and Molecular Biology, Uppsala University, Box 596, SE-751 24 Uppsala, Sweden.

[4]Department of Synthesis and Chemical Technology of Pharmaceutical Substances with Computer Modeling Laboratory, Faculty of Pharmacy, Medical University of Lublin, 4A Chodźki St., 20093 Lublin, Poland

[5]Division of Physiology, Pharmacology and Neuroscience, School of Life Sciences, Queen's Medical Centre, University of Nottingham, Nottingham, NG7 2UH United Kingdom.

[6]Centre of Membrane Proteins and Receptors, University of Birmingham and University of Nottingham, Nottingham, NG7 2UH, United Kingdom.

[7]Departments of Psychiatry and Pharmacology, College of Physicians and Surgeons, Columbia University, New York, NY 10032, United States

[8]Division of Molecular Therapeutics, New York State Psychiatric Institute, New York, NY 10032, United States

9Current address: OMass Therapeutics, ARC Oxford, OX4 2GX, United Kingdom

[10]UCB BioPharma SPRL, Chemin de Foriest, Braine-l'Alleud, B-1420, Belgium

[11]Current address: Kesmalea Therapeutics, 8 Bloomsbury Street, London, WC1B 3SR, United Kingdom

[12]Current address: Eli Lilly and Company, Lilly Biotechnology Center, 10290 Campus Point Dr, San Diego, CA 92121 USA

^ these authors contributed equally *Correspondence should be addressed to: Rob Lane; rob.lane@nottingham.ac.uk & Jens Carlsson; jens.carlsson@icm.uu.se

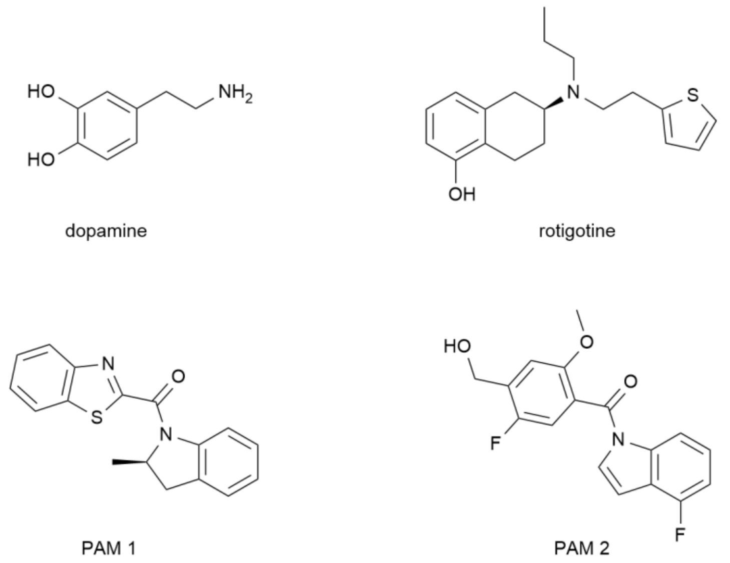

Supporting Information S1. Structures of the dopamine D2 receptor agonists (dopamine, rotigotine) and positive allosteric modulators (D2 PAM1, D2 PAM2) relevant for the presented study.

Supporting Information S2: The D2 PAM displays probe dependence. The ability of the PAM to modulate the action of various agonists at the D2LR expressed in Flp-In CHO cells was measured using a whole cell [^{3}H]raclopride binding assay or a functional assay measuring inhibition of forskolin stimulated cAMP production using a BRET biosensor. Values are expressed as mean ± S.E.M. from four separate experiments.

Orthosteric ligand	pK_A[a] (K_A, nM)	Logτ_A (τ_A)	pK_B[b] (K_b, μM)	Logα[c] (α)	Logαβ[d] (αβ)	Logβ[e] (β)
[^{3}H]raclopride	8.86 ± 0.16 (1.4)		5.26 ± 0.10 (5.50)	-0.39 ± 0.12 (0.41)	-	-
dopamine	5.49 ± 0.13 (3240)	1.14 ± 0.04 (13.8)	5.26 ± 0.10 (5.50)	1.33 ± 0.10 (21)	1.35 ± 0.10 (22)	0.02 ± 0.02 (1.0)
ropirinole	5.87 ± 0.18 (1350)	1.14 ± 0.08 (13.8)	5.06 ± 0.06 (8.71)	1.14 ± 0.11 (14)	1.67 ± 0.12 (47)	0.53 ± 0.05 (3.4)
apomorphine	7.08 ± 0.11 (83)	0.93 ± 0.02 (8.5)	5.20 ± 0.10 (6.31)	1.05 ± 0.11 (11)	1.21 ± 0.13 (16)	0.16 ± 0.07 (1.4)
aripiprazole	7.93 ± 0.04 (12)	0.03 ± 0.05 (1.7)	5.14 ± 0.04 (7.24)	0.43 ± 0.10 (2.7)	1.61 ± 0.14 (41)	1.18 ± 0.10 (15)
S-(3)-PPP	5.76 ± 0.18 (1740)	0.33 ± 0.05 (2.14)	5.29 ± 0.05 (5.12)	0.40 ± 0.11 (2.5)	1.46 ± 0.16 (28)	1.06 ± 0.11 (11)
bromocriptine	8.88 ± 0.15 (1.3)	0.66 ± 0.04 (4.6)	5.09 ± 0.13 (8.12)	0.27 ± 0.07 (1.9)	0.76 ± 0.13 (5.8)	0.49 ± 0.11 (3.1)
lisuride	9.51± 0.11	0.62 ± 0.05	5.25 ± 0.07	0.55 ± 0.04	0.66 ± 0.06	0.11 ± 0.04

	(0.3)	(4.2)	(5.62)	(3.5)	(4.6)	(1.3)
pergolide	7.60 ± 0.12<br>(25)	1.01 ± 0.03<br>(10.2)	5.08 ± 0.10<br>(8.32)	1.24 ± 0.14<br>(17)	1.50 ± 0.15<br>(32)	0.26 ± 0.05<br>(1.8)
pramipexole	5.88 ± 0.18<br>(1318)	1.05 ± 0.04<br>(11.2)	5.16 ± 0.11<br>(6.92)	1.21 ± 0.11<br>(16)	1.37 ± 0.12<br>(23)	0.16 ± 0.05<br>(1.4)
rotigotine	8.51± 0.32<br>(3.1)	1.17 ± 0.05<br>(14.8)	5.33 ± 0.14<br>(4.68)	1.37 ± 0.09<br>(23)	1.17 ± 0.09<br>(15)	-0.20 ± 0.04<br>(0.6)

The negative logarithm of the equilibrium dissociation constant of the orthosteric agonist/antagonist[a] and the D2 PAM[b] determined in the radioligand binding assay.
[c]Logarithm of the cooperativity factor between the D2 PAM and orthosteric ligand affinity determined in the radioligand binding assay.
[d]Logarithm of the net cooperativity factor between the D2 PAM and the orthosteric agonists determined in the functional assay
[e]Estimate of the logarithm of the modulatory factor and orthosteric ligand efficacy determined in the functional assay

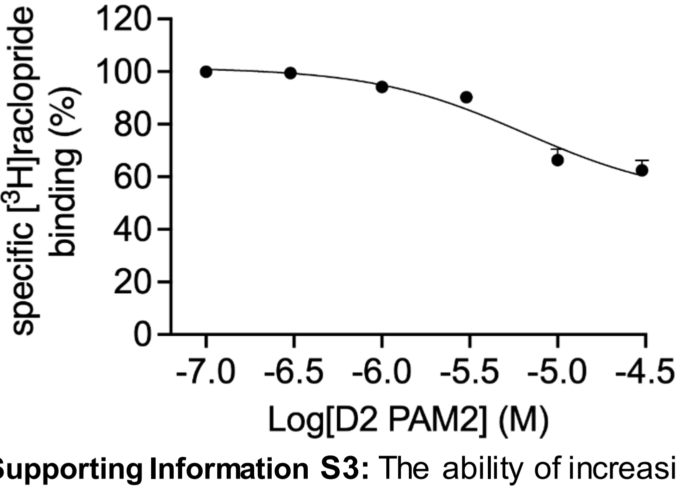

Supporting Information S3: The ability of increasing concentrations of the D2 PAM2 to modulate [^{3}H]raclopride binding in a whole cell radioligand binding assay. Data

represent the mean and S.E.M of 9 individual experiments performed in duplicate. These data were fitted to an allosteric ternary complex model to to derive a value of affinity (pK_b = 5.26 ± 0.15, K_b = 5.5 μM) and cooperativity Logα = -0.37 ± 0.05, α = 0.43).

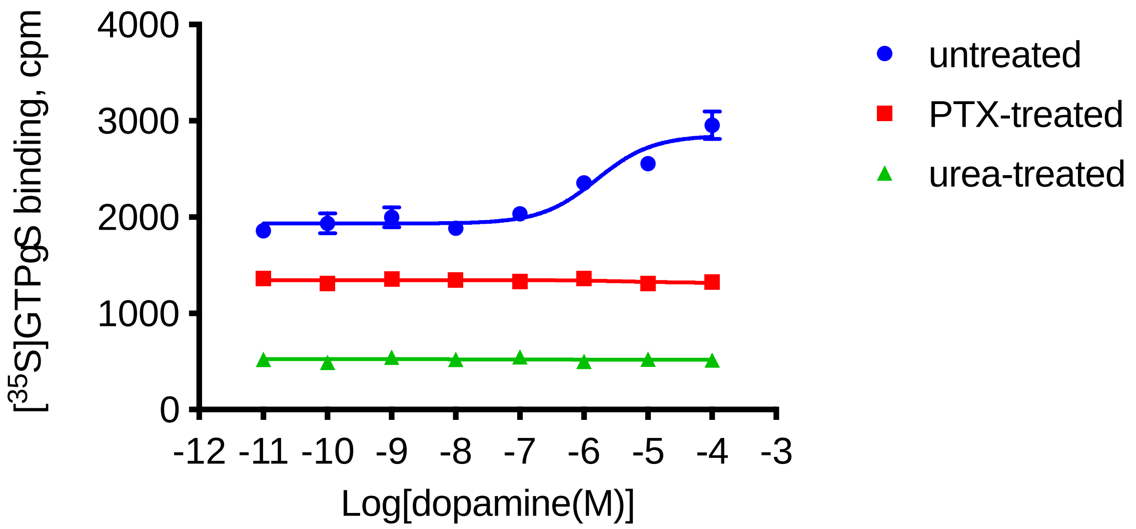

Supporting Information S4: [^{35}S]GTPγS binding reveals that treatment with Pertussis toxin (PTX) or urea stripping to remove heterotrimeric G proteins ablates the response of dopamine in membranes of FlpinCHO cells expressing the $hD_{2L}R$. Data are expressed as mean ± S.E.M. from three separate experiments.

Supporting Information S5: The D2 PAM increases the affinity of the radiolabeled agonist [^{3}H]rotigotine. The ability of the PAM to modulate the binding of [^{3}H]rotigotine to the $D_{2L}R$ expressed in Flp-In CHO cells was measured in saturation binding assays. Values are expressed as mean ± S.E.M. from four separate experiments.

	K_d (nM)[a]	B_{max} ($pmol•mg^{-1}$)
Control	0.72 ± 0.11	1.00 ± 0.18
+ 0.1mM GppNHp	6.40 ± 0.65*	1.37 ± 0.19
+ 10μM D2 PAM	0.31 ± 0.04	1.66 ± 0.23
+ 10μM D2 PAM + 100 mM NaCl	0.47 ± 0.05	1.71 ± 0.27
+ 10μM D2 PAM + 0.1 mM GppNHp	0.61 ± 0.15	1.45 ± 0.21

[a]The equilibrium dissociation constant of [^{3}H]rotigotine.
[b]The total density of [^{3}H]rotigotine binding sites.

Supporting Information S6: The D2 PAM increases the percentage of fluorescent agonist PPHT-d1 that dissociates in the slow phase. The ability of the PAM to modulate the binding of PPHT-d1 to the SNAP-D2sR expressed in Flp-In CHO cell membranes. Values are expressed as mean ± S.E.M. from four separate experiments.

	- Gpp(NH)P	+ 0.1 mM Gpp(NH)p							
[D2 PAM]	0	0	0.1 mM	0.3 mM	1 mM	3 mM	10 mM	30 mM	100 mM
K_{fast}	0.353 ± 0.003	0.346 ± 0.023							
K_{slow}	0.04 ± 0.000	0.075 ± 0.006							
% fast	60 ± 0.4	90 ± 1.6	87 ± 1.6	83 ± 2.2	83 ± 1.5	78 ± 1.5	68 ± 1.2	52 ± 0.8	60 ± 1.5

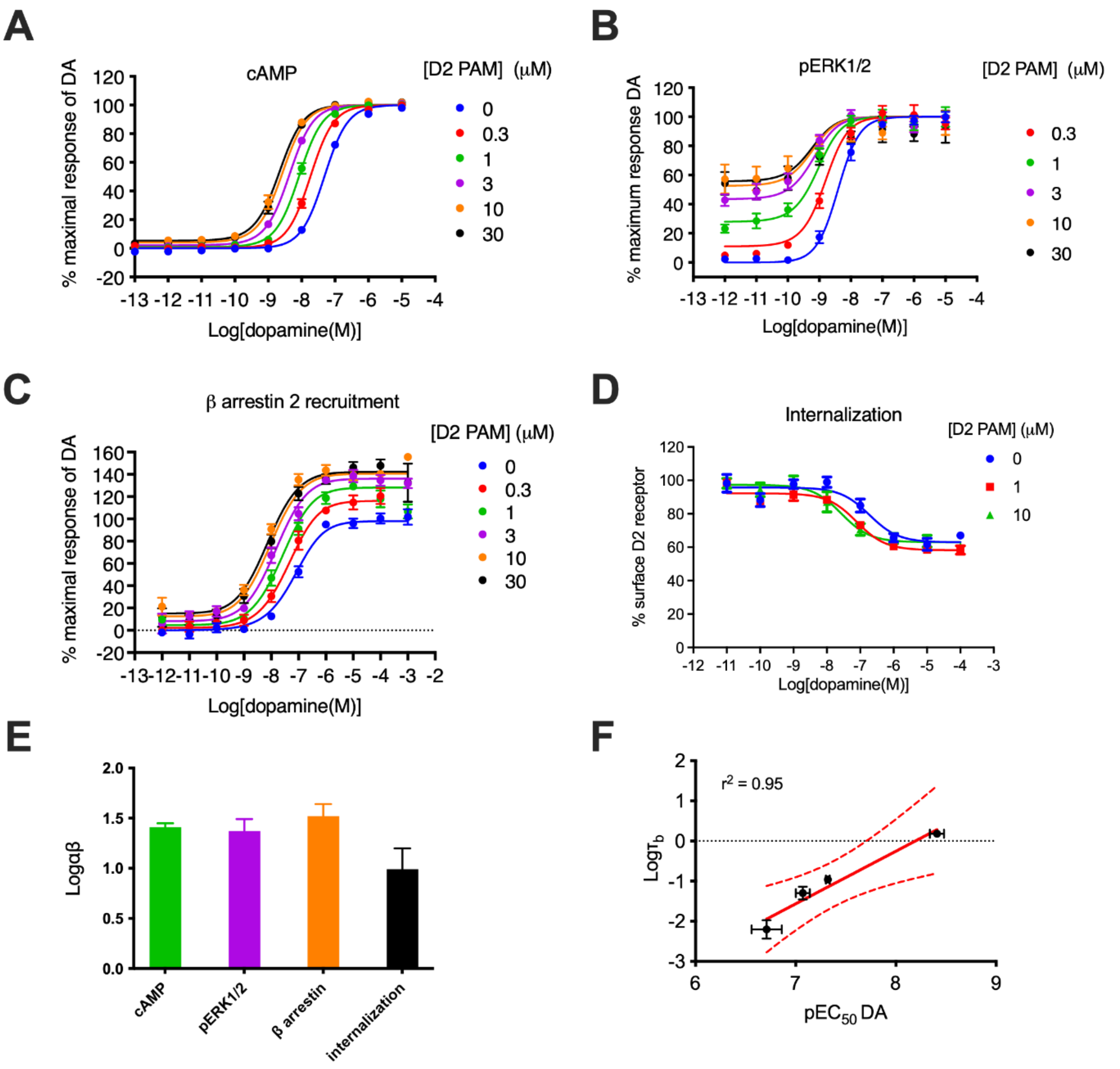

Supporting Information S7: The action of D2 PAM2 to modulate the action of dopamine at the D2R measured in an assay of $G_{i/o}$ inhibition of cAMP production (A), ERK1/2 phosphorylation (B), β arrestin 2 recruitment (C) and D2R internalization measured in a whole cell ELISA assay (D). These data were analyzed with an operational model of allostery to derive values of affinity and cooperativity displayed in supporting information S8. (E) There were no significant differences in the combined cooperativity factor between dopamine and D2 PAM2 across the different assays. (F) There was a correlation between the efficacy of D2 PAM2 ($\log \tau_b$) and the potency of dopamine (pEC_{50}) in each of the assays, Pearson r = 0.97, P = 0.02. These data could be fitted with a linear regression (with 95% CI), r^2 = 0.95.

Supporting information S8: Parameters describing the affinity, cooperativity with dopamine and intrinsic efficacy of D2 PAM2 across different signaling and regulatory endpoints.

	cAMP	pERK1/2	β arrestin recruitment	D_2R internalization
$pK_B{}^a$	5.37 ± 0.04	5.36 ± 0.13	5.18 ± 0.12	5.30 ± 0.29
$Log\alpha\beta^b$	1.41 ± 0.04	1.37 ± 0.12	1.52 ± 0.12	0.99 ± 0.21
$Log\tau_B{}^c$	-0.96 ± 0.04	0.18 ± 0.03	-1.30 ± 0.16	-2.20 ± 0.23

[a]The negative logarithm of the equilibrium dissociation constant of the D2 PAM2 determined in the functional assay
[b]Logarithm of the cooperativity factor between D2 PAM2 and dopamine determined in the functional assay
[C]The logarithm of the intrinsic efficacy of D2 PAM2 determined in the functional assay

Supporting information S9: The D2 PAM displays subtype selectivity. The ability of the PAM to modulate the action of various agonists at the $D_{2L}R$, D_3R and $D_{4.4}R$ expressed in Flp-In CHO cells The ability of the PAM to modulate the action of various agonists at the $D_{2L}R$ expressed in Flp-In CHO cells was measured using a cell membrane [^{3}H]raclopride ($D_{2L}R$, D_3R) or [^{3}H]spiperone ($D_{4.4}R$) binding assay. Values are expressed as mean ± S.E.M. from four separate experiments.

	$D_{2L}R$	D_3R	$D_{4.4}$ R
$pK_A{}^a$ (K_A, mM)	5.44 ± 0.03 (3.6)	6.88 ± 0.04 (0.13)	6.31 ± 0.03 (0.49)
$pK_B{}^b$ (K_B, mM)	4.65 ± 0.05 (22)	4.77 ± 0.14 (17)	-
$\log\alpha$_[^{3}H]raclo[c] (α_[^{3}H]raclo)	-0.19 ± 0.05 (0.64)	-0.14 ± 0.04 (0.72)	-
$\log\alpha$_DA[d] (α_DA)	1.30 ± 0.02 (20)	0.98 ± 0.02 (9.5)	-

The negative logarithm of the equilibrium dissociation constant of the orthosteric agonist/antagonist[a] and the D2 PAM[b] determined in the radioligand binding assay.
Logarithm of the cooperativity factor between the D2 PAM and DA[c] or the radioligand[d] determined in the radioligand binding assay.

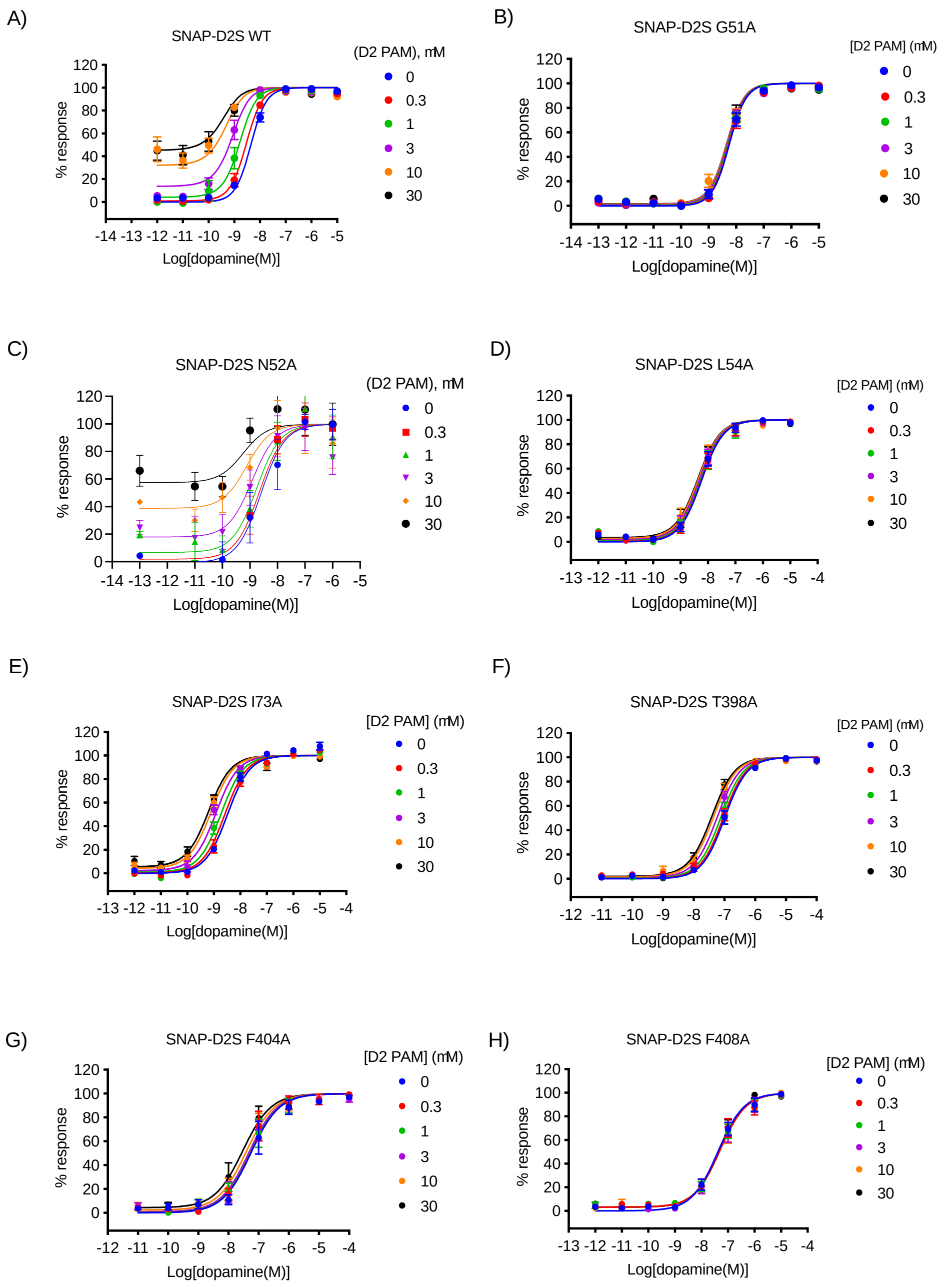

Supporting information S10: The mutation of residues within an intracellular pocket of the D2R abrogates the action of the D2 PAM. The ability of the D2 PAM to potentiate the functional action of DA in an assay measuring the inhibition of forskolin stimulated intracellular cAMP accumulation in Flp-In CHO cells expressing the SNAP-D2SR WT (A) the mutant F50$^{1.48}$A (B), N52$^{1.50}$A (C), L54$^{1.52}$A (D), I73$^{2.43}$A (E), T398$^{7.54}$A (F), F404$^{8.50}$A (G), F408$^{8.54}$A (H). Data represents mean ± standard error of four individual experiments performed in duplicate. Data were fitted with an operational model of allostery to derive values of affinity, cooperativity with dopamine and efficacy as displayed in Table 3.

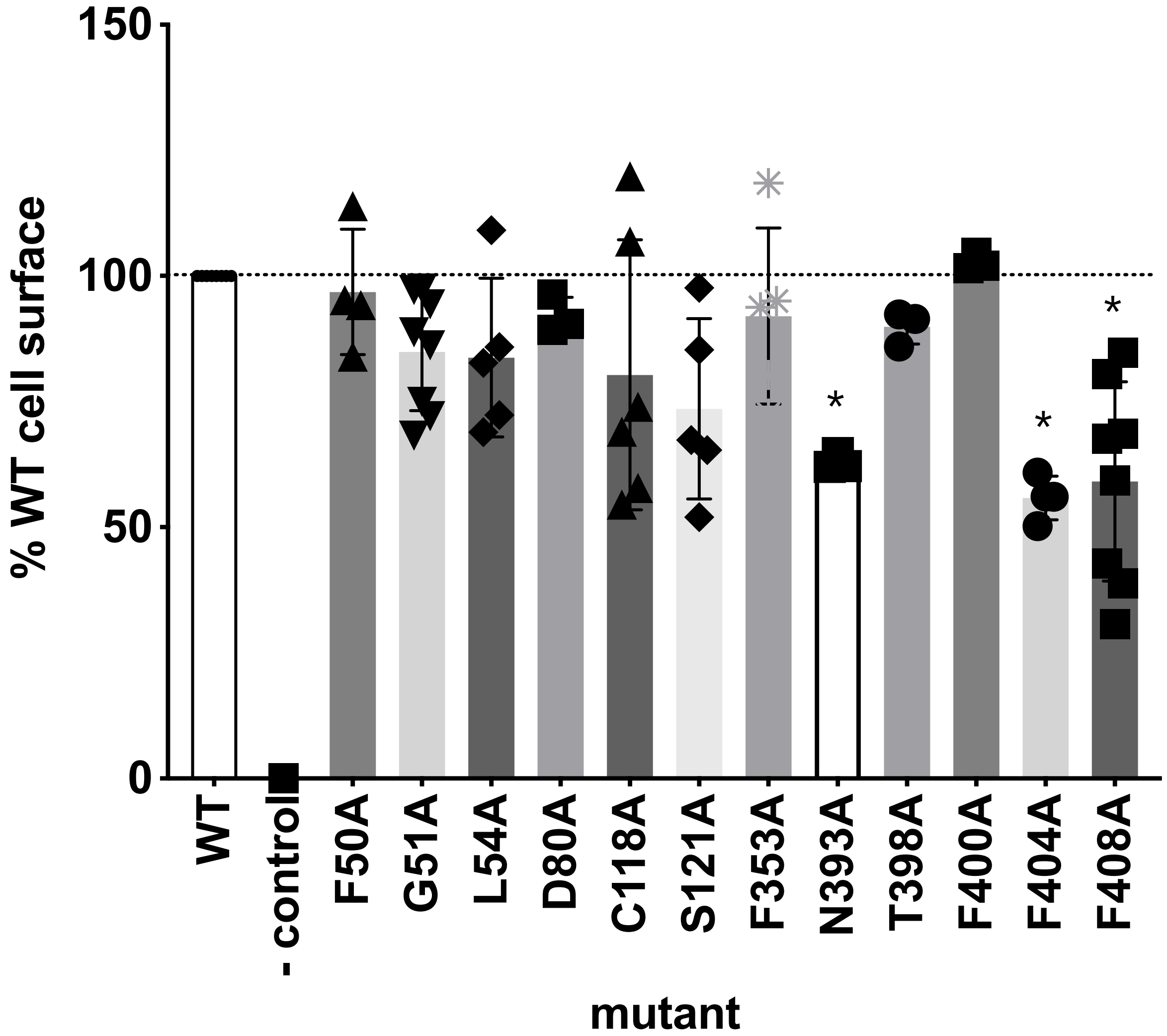

Supporting information S11: ELISA assessment of cell surface expression. Values are expressed as mean ± S.E.M. from three separate experiments.

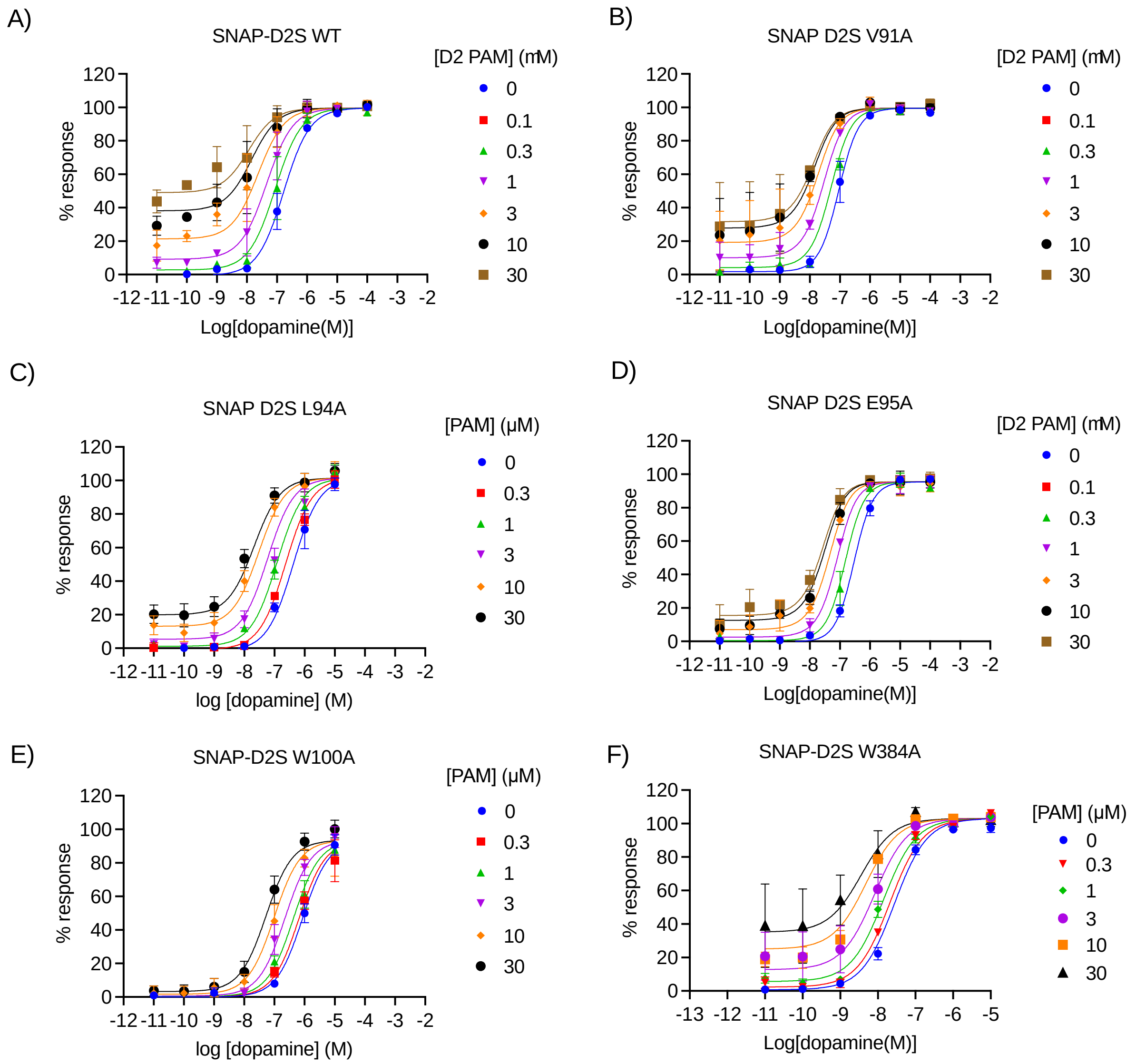

Supporting information S12: The effect of D2 PAM is not affected by mutation of residues within a previously proposed extracellular binding pocket. The ability of the D2 PAM to potentiate the functional action of DA in an assay measuring the inhibition of forskolin stimulated intracellular cAMP accumulation in FlpInCHO cells expressing the SNAP-D$_{2S}$R WT (A) the mutant V91$^{2.61}$A (B), D80$^{2.64}$A (C), E95$^{2.65}$A (D), W100^{ECL1}A (E), W384$^{7.40}$A (F). Data represents mean ± standard error of four individual experiments performed in duplicate. Data were fitted with an operational model of allostery to derive values of affinity, cooperativity with dopamine and efficacy as displayed in Supplementary Table 5.

Supporting information S13. The effect of D2 PAM is not affected by mutation of residues within a previously proposed extracellular binding pocket. The affinity of the D2 PAM, its intrinsic efficacy and cooperativity with DA at WT or mutant SNAP-$D_{2S}R$ expressed in Flp-In CHO cells was measured in a functional cAMP assay using a BRET biosensor. Values are expressed as mean ± S.E.M. from four separate experiments.

	pK_B[c] (K_B, μM)	Logαβ[d] (αβ)	Logτ[e] (τ)
WT	4.93 ± 0.22 (11.7)	1.60 ± 0.25 (39.8)	0.14 ± 0.10 (1.38)
V91$^{2.61}$A	5.59 ± 0.21 (2.6)	0.93 ± 0.18 (8.5)	-0.23 ± 0.08 (0.59)
L94$^{2.64}$A	4.90 ± 0.12 (12.6)	1.54 ± 0.10 (34.7)	-0.43 ± 0.07 (0.37)
E95$^{2.65}$A	5.49 ± 0.12 (3.2)	1.07 ± 0.10 (11.7)	-0.46 ± 0.07 (0.35)
W100^{ECL1}A	4.93 ± 0.28 (11.7)	1.54 ± 0.10 (34.7)	-0.43 ± 0.06 (0.37)
W384$^{7.40}$A	4.86 ± 0.24 (13.8)	1.04 ± 0.15 (11.0)	-0.24 ± 0.08 (0.57)

[a]The negative logarithm of the equilibrium dissociation constant of DA in the radioligand binding assay
[b]The potency of DA in the functional assay
[c]The negative logarithm of the equilibrium dissociation constant of the D2 PAM determined in the functional assays
[d]Logarithm of the net cooperativity factor between the D2 PAM and DA determined in the functional assay
[e]Estimate of the logarithm of the modulatory factor and orthosteric ligand efficacy determined in the functional assay

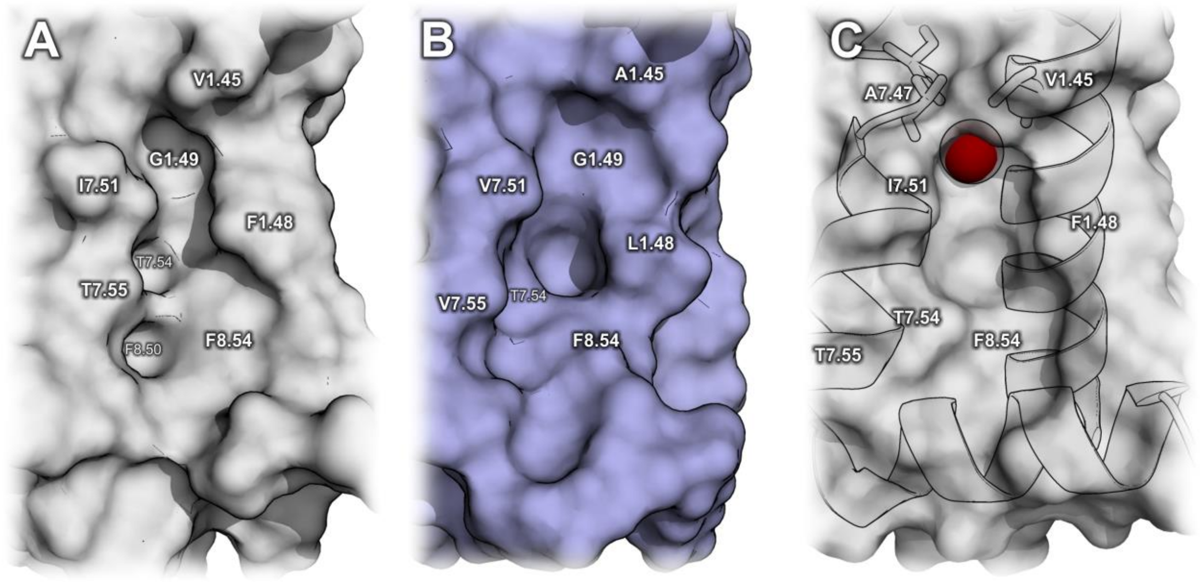

Supporting information S14: Comparison of allosteric pocket in different dopamine receptors. (A) and (B) Differences observed in cryo-EM structures of the dopamine D_2R (A) and D_4R (B) in their active states deposited in the PDB database (6VMS and 8IRU, respectively). Visual inspection of 6VMS PDB structure reveals a deep cavity in D_2R, potentially capable of hosting a forked planar substituent, resembling the shape of m-hydroxymethyl-methoxyphenyl moiety of D2 PAM2. (C) X-ray structure of D_2R with a water molecule (dark-red sphere) bound in the middle of the transmembrane region between helices 1 and 7.

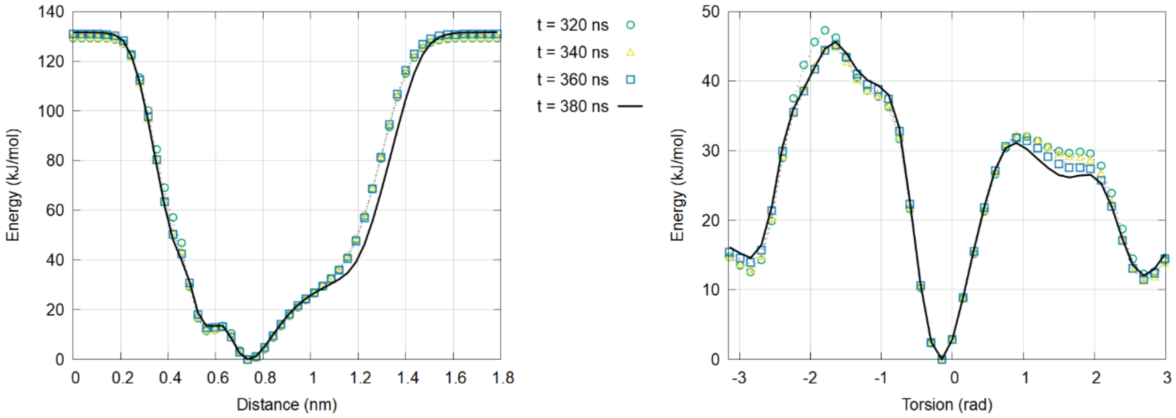

Supporting information S15: Convergence of the metadynamics simulations. Two-dimensional free energy profiles along the distance or the torsion axis collected every 20 ns show that no relevant changes to the free energy landscape occur over the last 60 ns of the simulation.

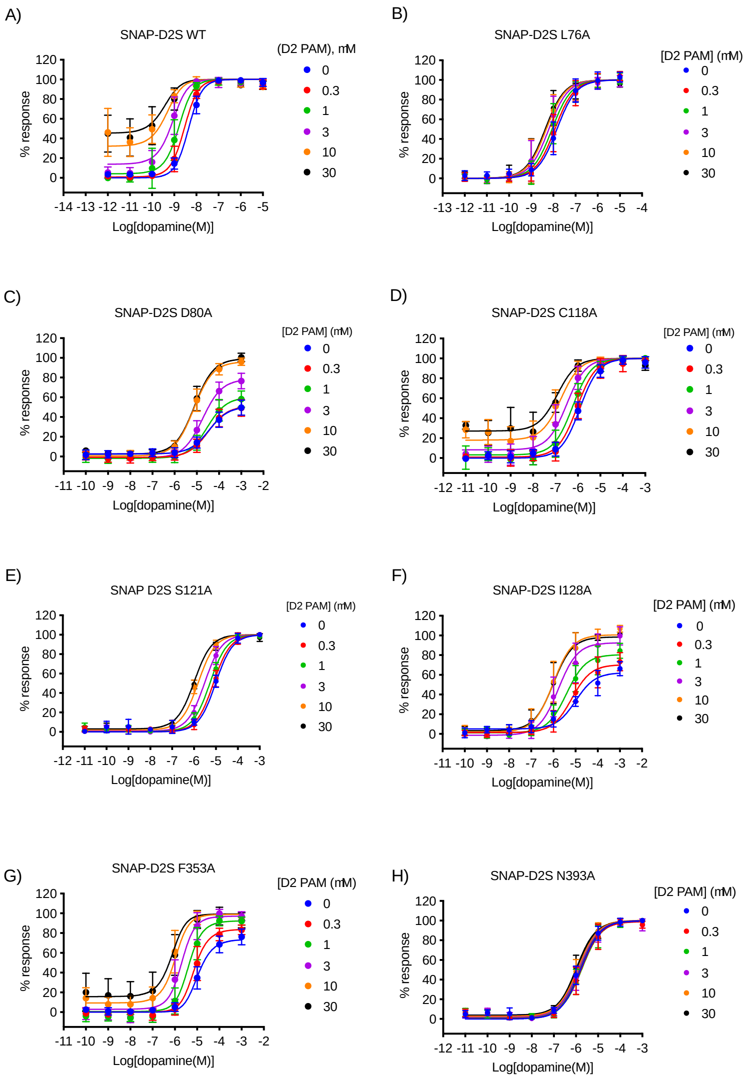

Supporting information S16: The effect of D2 PAM2 on DA binding is inhibited by mutation of residues involved in receptor activation. The ability of the D2 PAM to potentiate the functional action of DA in an assay measuring the inhibition of forskolin stimulated intracellular cAMP accumulation in FlpInCHO cells expressing the SNAP-D2sR WT (A) the mutant L76$^{2.46}$A (B), D80$^{2.50}$A (C), C118$^{3.36}$A (D), S121$^{3.39}$A (E), I128$^{3.46}$A (F), F353$^{6.44}$A (G) and N393$^{7.49}$A (H). Data represents mean ± S.E.M of four individual experiments performed in duplicate. Data were fitted with an operational model of allostery to derive values of affinity, cooperativity with dopamine and efficacy as displayed in Table 3.

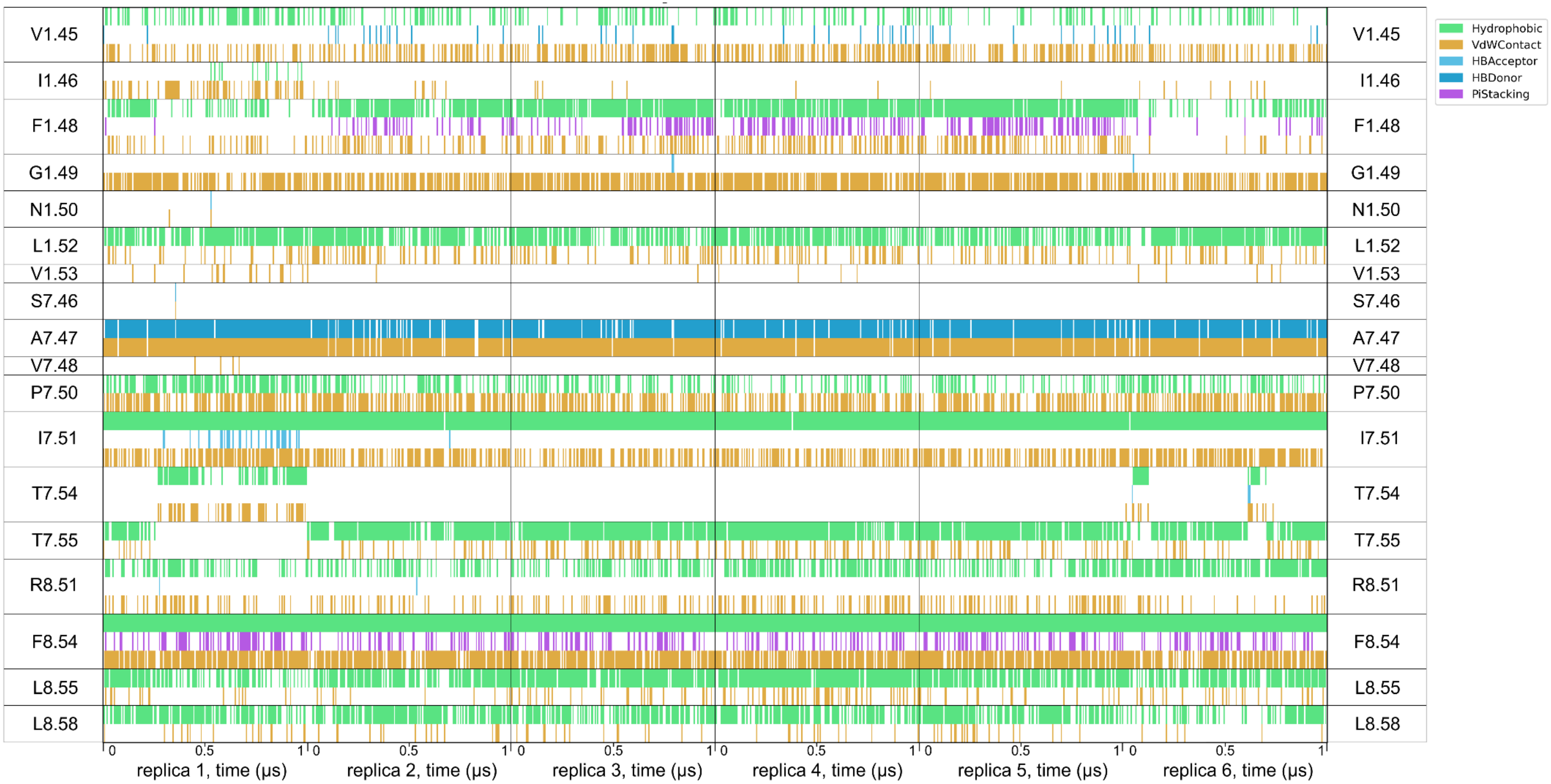

Supporting information S17: Protein-modulator interaction barcodes in rotigotine- and PAM2-bound receptor simulations. Interactions between D2 PAM2 and the receptor protein within MD trajectories of the PAM/rotingotine/receptor complex were calculated using ProLIF. The most stable interactions are hydrogen bonding and van der Waals contacts with A7.47, hydrophobic interactions with L1.52, I7.51 and F8.54. Several stacking interactions with F1.48 and F8.54 are also worth noting.

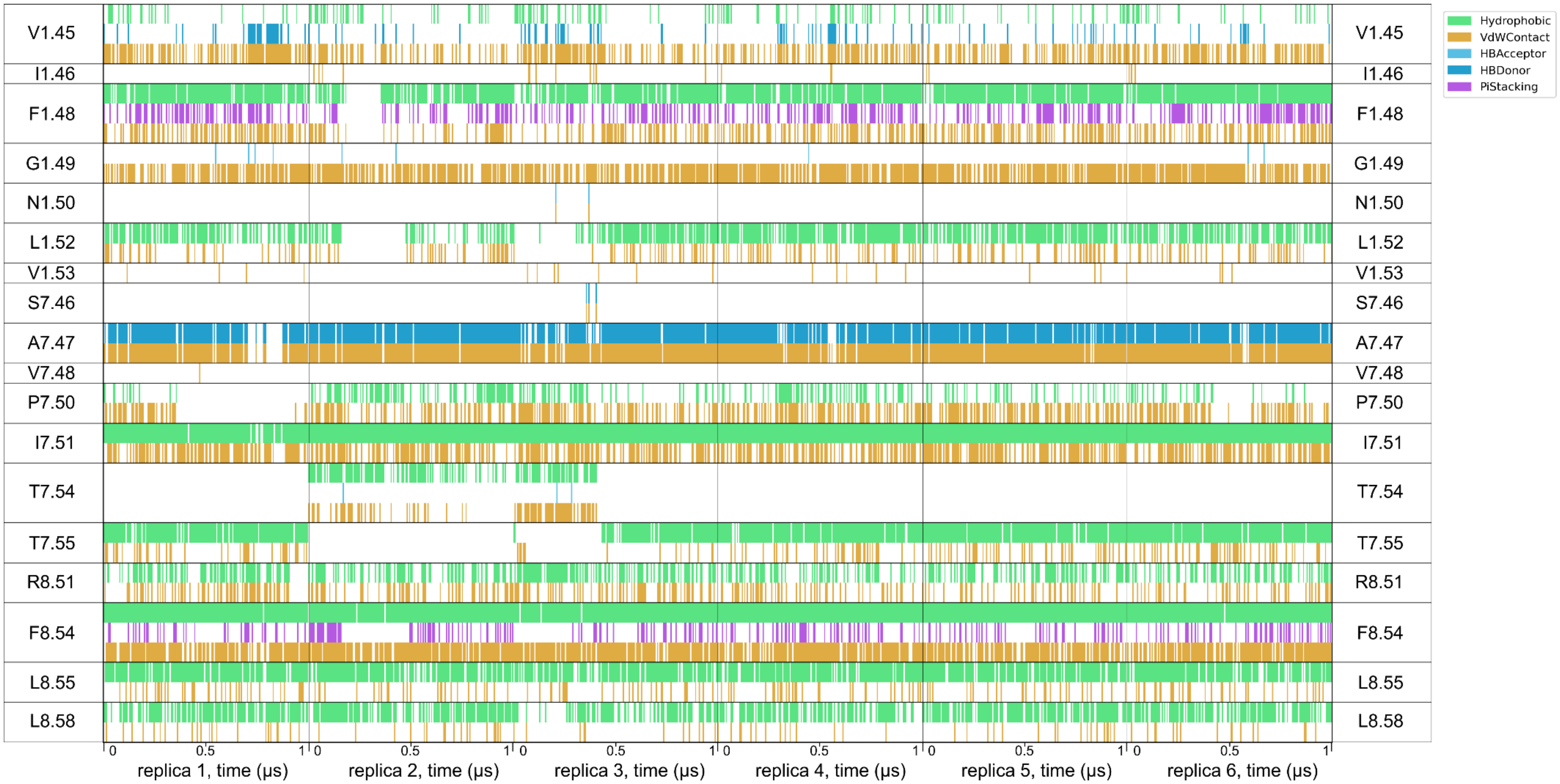

Supporting information S18: Protein-modulator interaction barcodes in PAM2-bound receptor simulations. Interactions between D2 PAM2 and the receptor protein within MD trajectories were calculated using ProLIF. The most stable interactions are hydrogen bonding and van der Waals contacts with A7.47 and hydrophobic interactions with I7.51 and F8.54. Several stacking interactions with F1.48 and F8.54 are also worth noting.

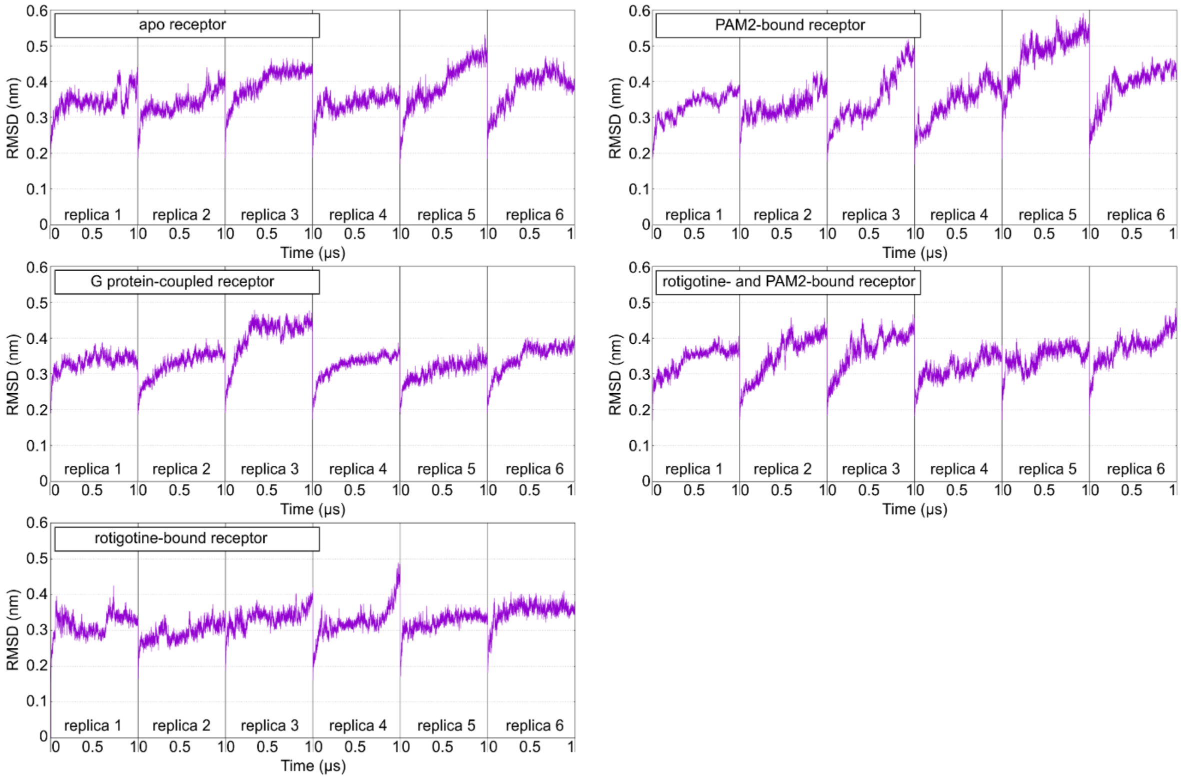

Supporting information S19: RMSD of the heavy atoms of the receptor protein in all simulations. The drift apparent in most simulations is linked to conformational changes induced by removal of G protein from the active-state receptor. Presence of either G protein or rotigotine limits the drift from the active-state starting structure.

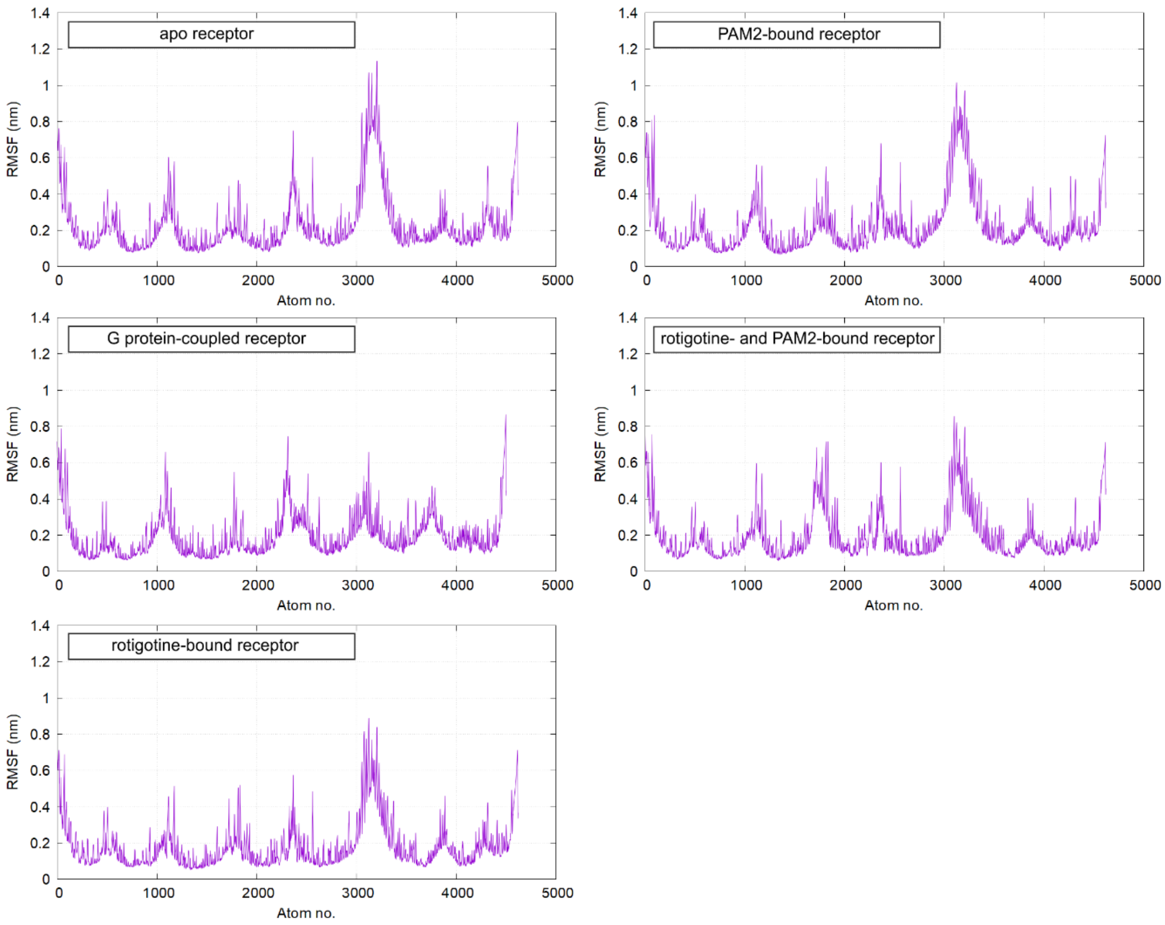

Supporting information S20: RMSF of the receptor protein in all simulations. The notable peak at atom index range 3000-3300 corresponds to mobility of ICL3 and the intracellular end of TM6. The peak appearing in 'rotigotine- and PAM2-bound receptor' panel in the atom index range 1600-1900 corresponds to mobility of ICL2.
